# Spatially identifying regions of tumor recurrence in patients with suspected recurrent glioma using physiologic MRI and machine learning

**DOI:** 10.1038/s41746-026-02823-y

**Published:** 2026-06-25

**Authors:** Jacob Ellison, Nate Tran, Tracy L. Luks, Paramjot Singh, Angela Jakary, Tiffany Ngan, Julia Cluceru, Joanna J. Phillips, Yan Li, Annette M. Molinaro, Valentina Pedoia, Anny Shai, Devika Nair, Javier E. Villanueva-Meyer, Mitchel S. Berger, Shawn L. Hervey-Jumper, Manish Aghi, Susan M. Chang, Janine M. Lupo

**Affiliations:** 1 Department of Radiology and Biomedical Imaging, UCSF, San Francisco, CA USA; 2 Center for Intelligent Imaging, UCSF, San Francisco, CA USA; 3 UCSF-UC Berkeley Graduate Program in Bioengineering, San Francisco and Berkeley, CA USA; 4 Department of Neurological Surgery, University of California, San Francisco, San Francisco, CA USA; 5 Department of Pathology, University of California, San Francisco, San Francisco, CA USA

**Keywords:** Cancer, Computational biology and bioinformatics, Medical research, Neurology, Neuroscience, Oncology

## Abstract

Despite prior success in classifying recurrent glioma noninvasively with multi-parametric MRI and AI, clinical applicability has yet to be demonstrated due to a lack of robust model evaluation and spatial preservation of tumor characteristics. This study develops, robustly evaluates, and clinically validates an interpretable model for predicting recurrent tumors from spatially varying, histopathologically-confirmed tissue samples. Machine learning models were developed using 254 pre-surgical multi-parametric MRI patches surrounding coordinates of tissue samples taken during recurrent surgery. A test AUROC of 0.74 ± 0.08 for distinguishing recurrent tumors, and 0.99 ± 0.01 for normal-appearing brain, demonstrated the feasibility of spatially mapping heterogeneity. Important features were consistent with current literature, and uncertainty was correlated with model failures (*p* ≤ 0.05). Volumetrics derived from prediction maps of recurrent tumors generated using a separate cohort of 56 patients with recurrent high-grade gliomas were significantly associated with survival. These results demonstrate a step towards clinical applicability of spatially mapping glioma recurrence.

## Introduction

Diffuse gliomas are the most common primary malignant brain tumor, with an incidence rate of approximately 20,000 every year in the US and an average 5-year survival rate of only 5% for the most aggressive Isocitrate Dehydrogenase (IDH)-wildtype glioblastoma subtype (GBM)^1^. After maximal surgical resection, treatment with temozolomide and other chemotherapeutic agents and radiation therapy is the current standard of care for patients with GBM and higher-grade IDH-mutant astrocytomas to help impede the growth of residual infiltrating tumor cells^2^^,3^. Despite this aggressive treatment, virtually all patients with GBM recur within 7–10 months and have a median survival time following progression of only 6–7 months^1,4–6^. Tumor recurrence in patients with high-grade glioma (HGG) is difficult to diagnose because treatment-induced injury—typically characterized by a combination of apoptosis, gliosis, and inflammation—can often mimic tumor recurrence on conventional anatomical MRI^7^. The appearance of these treatment effects (TxE) has become even more prevalent with the recent advent of immuno- and other targeted therapies^8^, where the risks are even higher. If recurrence is incorrectly diagnosed, a patient may be removed from an effective therapy, which could invalidate the results of a clinical trial or expose a patient to unnecessary surgical or medical intervention. Hence, accurate identification of the exact location and extent of recurrent tumors within newly enlarging lesions is critical for effective patient management.

Highly trained neuro-radiologists have difficulty distinguishing between recurrent tumor (rTumor) and TxE from anatomical MRI, with at most 66% accuracy^9^. Tissue sampling and histopathological analysis, the gold standard of rTumor assessment, pose a risk to patient wellbeing and quality of life and involve selecting only a few samples, potentially missing the region of tumor recurrence. Noninvasive lesion characterization is also an ongoing challenge for monitoring response to treatment in general^10,11^, with the current Response Assessment in Neuro-Oncology criteria for HGG (RANO 2.0)^12^ still relying solely on standard T1- and T2-weighted MR imaging, despite these known pitfalls.

While PET has shown similar success to MRI in identifying recurrent tumors from treatment-related abnormalities, there is no clear evidence of diagnostic superiority for either method^13^, and RANO states the acceptance of PET as an adjunct to MRI^14^. Emerging data suggest that incorporating more advanced MRI techniques may be useful for probing underlying biological differences: i.e., diffusion-weighted imaging for capturing the restricted water movement from the density of proliferating tumor cells, whereas perfusion-weighted imaging evaluates the increased vasculature recruited to support a growing mass. The majority of these studies, however, involve calculating the mean diffusion- or perfusion-derived parameter value from an anatomic region of interest (ROI) and normalizing that value against that obtained from contralateral normal-appearing white matter in order to obtain a threshold that can distinguish TxE from true tumor recurrence. These ROI-based methods suffer from widely varying cutoff values for parameters due to interobserver variability, intratumoral heterogeneity, and the coexistence of treatment-induced changes and recurrent tumor within the same lesion. Such studies also typically use radiographic observations or a single tissue sample for outcome determination, ascribing a single diagnosis to a mixture of tissue types that could severely limit the accuracy of the diagnosis. Still, to date, relative cerebral blood volume (rCBV) maps from Dynamic Susceptibility Contrast (DSC) perfusion-weighted MRI have shown the most promise in identifying rTumor within the contrast-enhancing lesion (CEL)^15–18^. And although rCBV values from image-guided tissue samples have been reported to distinguish TxE from contrast-enhancing rTumor in smaller, correlative studies^15–18^, rCBV alone cannot identify recurrent tumor cells in the non-enhancing portion of the lesion^19^.

More recently, noninvasively distinguishing rTumor from TxE with MRI has been attempted using machine learning and deep learning methods^20–28^. These approaches have focused on classifying the lesions without optimizing for intra-tumoral heterogeneity, have mostly used features derived from anatomical images alone, used few training samples, and have varied clinical definitions of TxE. Because recurrent lesions often contain mixtures of tumor and non-tumoral compartments, including treatment-related changes, necrosis, gliosis, and cytotoxic edema, lesion-level assignment of TxE can be ambiguous. We therefore aimed to spatially distinguish recurrent tumors from all other types of lesional tissue, the majority of which were due to the effects of treatment. Spatial identification of rTumor in this framework aligns with surgical and therapeutic decision-making and may be more clinically applicable due to the wide variation of tissue characteristics that exist within these lesions and are not related to the presence of tumor. Classification of rTumor at sub-lesion resolution would preserve spatial information on the tumor heterogeneity^19^ and allow for more precise diagnosis of tumor recurrence, potentially guiding surgical resection to distinct locations with high probability of tumor cells outside of the CEL or preventing invasive surgery.

The goal of this study was to leverage our unique dataset of pathologically confirmed tissue samples with known coordinates on pre-surgical, multi-parametric anatomical and physiological MRI, along with machine learning, to spatially map areas of recurrent tumor in patients with glioma about to undergo surgery for a suspected recurrence. We hypothesize that framing this learning task as spatially classifying histopathologically confirmed tissue and using pre-surgical multi-parametric MR images will enable more precise identification of rTumor and avoid complications of tissue heterogeneity that confound most lesion-level analyses. Additionally, we expect that: (1) incorporating diffusion-weighted and DSC perfusion-weighted MRI into our models, which have been previously shown to provide value in identifying rTumor^7,17,29^^,30^, will significantly improve performance over anatomical imaging alone; (2) ensembling models across multiple subsets of the data will improve model generalizability; and (3) incorporating normal appearing brain data into model training will allow for the expanding of spatial map generation in inference beyond the T2-hyperintese lesion (T2L). To facilitate model interpretability, we quantified measures of prediction variance and entropic uncertainty^31^ from the ensembles to characterize model failures, and implemented permutation feature importance^32^ for interpreting the impact of each imaging feature. The clinical utility of our model was then further validated by conducting a multivariate survival analysis in a separate cohort that utilized the whole brain spatial maps of rTumor, enabled by tiered classification of abnormal brain, to extract the proportion of rTumor within the lesion.

## Results

### Classification of recurrent tumor

**Fig. 1:**
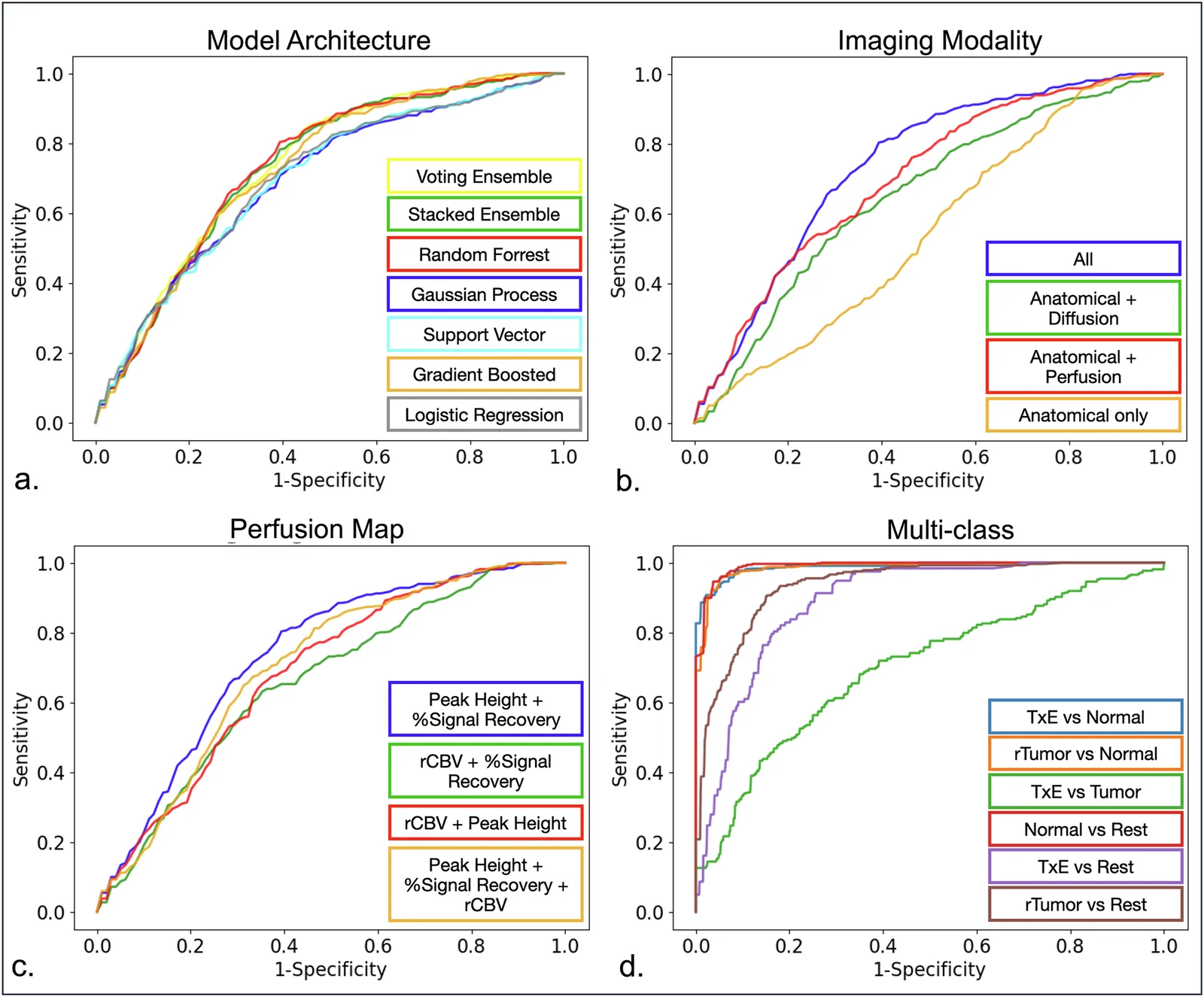
Receiver Operating Characteristic curves for each classification model. Classification performance for binary classification with different architectures (**a**), varied imaging modalities (**b**), using different perfusion imaging metrics (**c**), and multi-class classification (**d**) are shown. In the multi-class evaluation, One vs. Rest (OVR) and One vs. One (OVO) performance are shown.

The best binary classification model, with a testing AUROC of 0.74 ± 0.08, was the soft voting ensemble with voting weights of [3,3,0,1,3] for [support vector machine (SVM), random forest (RF), logistic regression (LR), Gaussian process (GP), gradient boosted (GB)] models, respectively (Fig. 1a, Table 1). Including diffusion significantly improved performance beyond anatomical imaging alone (Fig. 1b), with the best performance observed when normalized Peak-height (PH) and Percent signal recovery (%Recov) maps from DSC perfusion were included as inputs. When rCBV was included as an input in place of PH, model performance dropped significantly (Fig. 1c, Table 1), but when it was included in addition, the performance drop was nonsignificant. Although the multi-class classification model achieved excellent performance in distinguishing abnormal from normal appearing brain with a mean testing AUROC of 0.99 ± 0.01, it failed to improve the classification performance between Non-recurrent Tumor (NrT) and rTumor (0.72 ± 0.06 vs 0.74 ± 0.08; *p* = 0.35; Fig. 1d). As shown by the one vs. rest and one vs. one receiver operating characteristic (ROC) curves for each of the multi-class classification in Fig. 1d, the multi-class model also had excellent performance for separately distinguishing rTumor and NrT from normal-appearing brain (both 0.99 ± 0.01). Classification performance of NrT and rTumor across repetitions of testing using Youden’s J-Index for optimal thresholding and using a threshold of 0.5 are shown in Supplementary Fig. 1. Binary classification using the Random Forrest model on 5 disjoint test sets covering all the patients (without repetition) had an AUROC of 0.78 ± 0.06, as shown in Supplementary Fig. 4.

**Table 1:** Model comparisons across imaging modalities, perfusion metrics, and model types

	AUROC	% change from best	*p* value
**Imaging modalities**			
*All*	0.737 ± 0.069	-	-
*Anatomical + Perfusion*	0.701 ± 0.077	4.88%	0.131
*Anatomical + Diffusion*	0.650 ± 0.067	11.8%	0.0003
*Anatomical Only*	0.544 ± 0.051	26.2%	<0.0001
**Perfusion modalities**			
*Peak Height* + *% Recovery*	0.737 ± 0.069	-	-
*rCBV* + *% Recovery*	0.661 ± 0.052	10.3%	0.0005
*rCBV* + *Peak Height*	0.689 ± 0.067	6.51%	0.034
*Peak Height* + *% Recovery* + *rCBV*	0.705 ± 0.058	4.34%	0.130
**Model types**			
*Voting Ensemble*	0.738 ± 0.078	-	-
*Stacked Ensemble*	0.733 ± 0.068	0.68%	0.858
*Random Forest*	0.737 ± 0.069	0.14%	0.985
*Gaussian Process*	0.696 ± 0.078	5.69%	0.106
*Support Vector*	0.697 ± 0.082	5.56%	0.127
*Gradient Boosted*	0.728 ± 0.077	1.36%	0.708
*Logistic Regression*	0.701 ± 0.081	5.01%	0.162

### Model evaluation and failure characterization

**Fig. 2:**
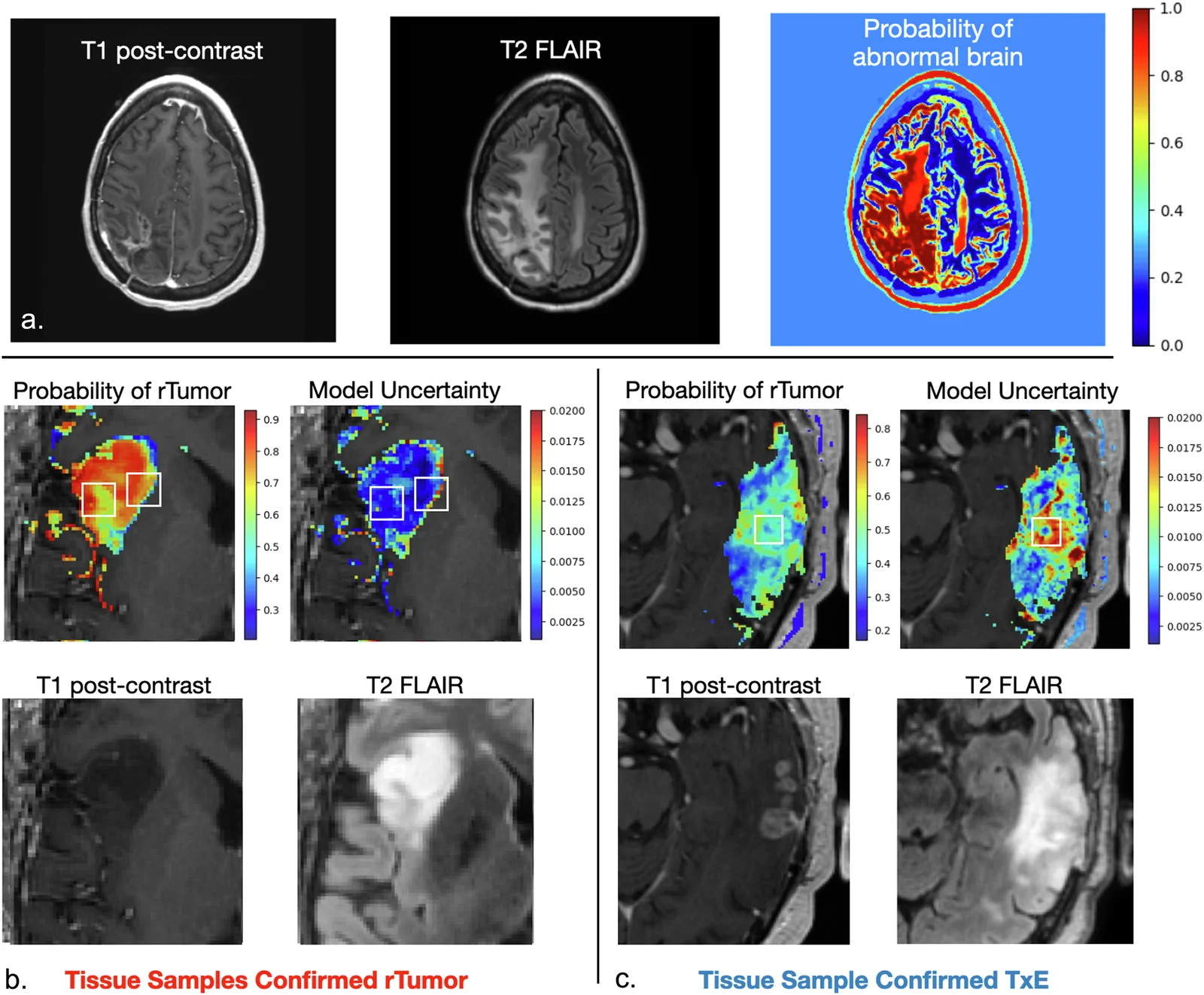
Spatial maps of recurrent tumor and model uncertainty. **a** Maps of predicted abnormal brain facilitate expansion of predictions to the whole brain. Spatial maps of ensemble probabilities of recurrent tumor (rTumor) and epistemic uncertainty for representative confirmed recurrent tumor tissue samples (**b**) and a tissue sample with confirmed treatment effect (TxE) (**c**). Maps qualitatively align with histopathologically confirmed tissue samples in bounding boxes in white. The maps are generated for test cases by the ensemble with the highest testing performance. Uncertainty maps could provide additional confidence for prediction success in rTumor.

Aleatoric, epistemic, and total uncertainty (defined in Eq. 1), as well as ensemble variance, were all significantly positively correlated (*p* ≤ 0.05) to model failures in the prediction of rTumor, demonstrating a higher confidence in the model’s predictions of rTumor compared to NrT, despite the less prediction failures observed for the NrT class. A negative trend was observed between epistemic uncertainty and prediction failures of NrT (*p* = 0.067; Table 2 and Supplementary Figs. 2 and 3). The resulting spatial maps qualitatively aligned well with confirmed pathology, with regions of higher (or lower) probabilities of rTumor on the spatial maps that were confirmed by histopathology also having lower uncertainty values, as illustrated in Fig. 2.

**Table 2:** Ensemble variance and uncertainty metrics for prediction failures

	Total uncertainty	Epistemic uncertainty	Aleatoric uncertainty	Ensemble variance
Correlation to failure in predicting rTumor	2.896 (*p* = 0.004)	24.91 (*p* = 0.004)	2.892 (*p* = 0.005)	63.83 (*p* = 0.002)
Correlation to failure in predicting NrT	0.480 (*p* = 0.100)	−1.84 (*p* = 0.067)	0.532 (*p* = 0.074)	−2.923 (*p* = 0.172)

### Feature importance analysis

**Fig. 3:**
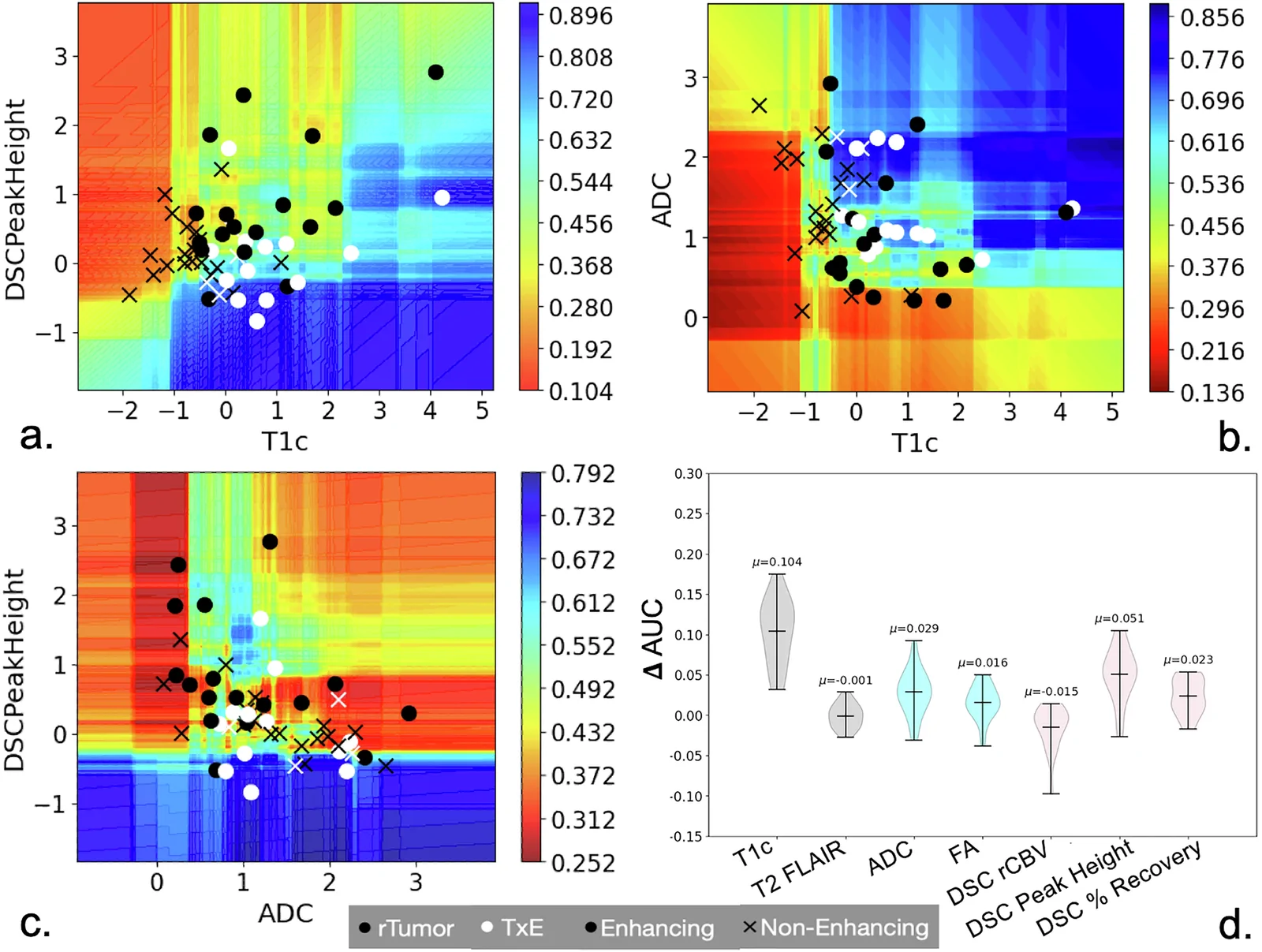
Important imaging features determined by permutation feature importance. **a**–**c** Decision boundaries fit along each pair of the three imaging features with the greatest impact to AUROC (T1-post-contrast (T1c), Apparent Diffusion Coefficient (ADC), and Dynamic Susceptibility Contrast Perfusion peak height (DSC PH)) for the best performing model (Voting Ensemble). **a** Decision boundary along DSC PH and T1c, **b** T1c and ADC. and **c** DSC PH and ADC. **d** Change in AUROC upon permuting each feature for a Random Forest fit on all seven imaging modalities including DSC relative Cerebral Blood Volume (rCBV). Non-enhancing samples show different signatures for non-recurrent Tumor (NrT) and recurrent tumor (rTumor).

**Fig. 4:**
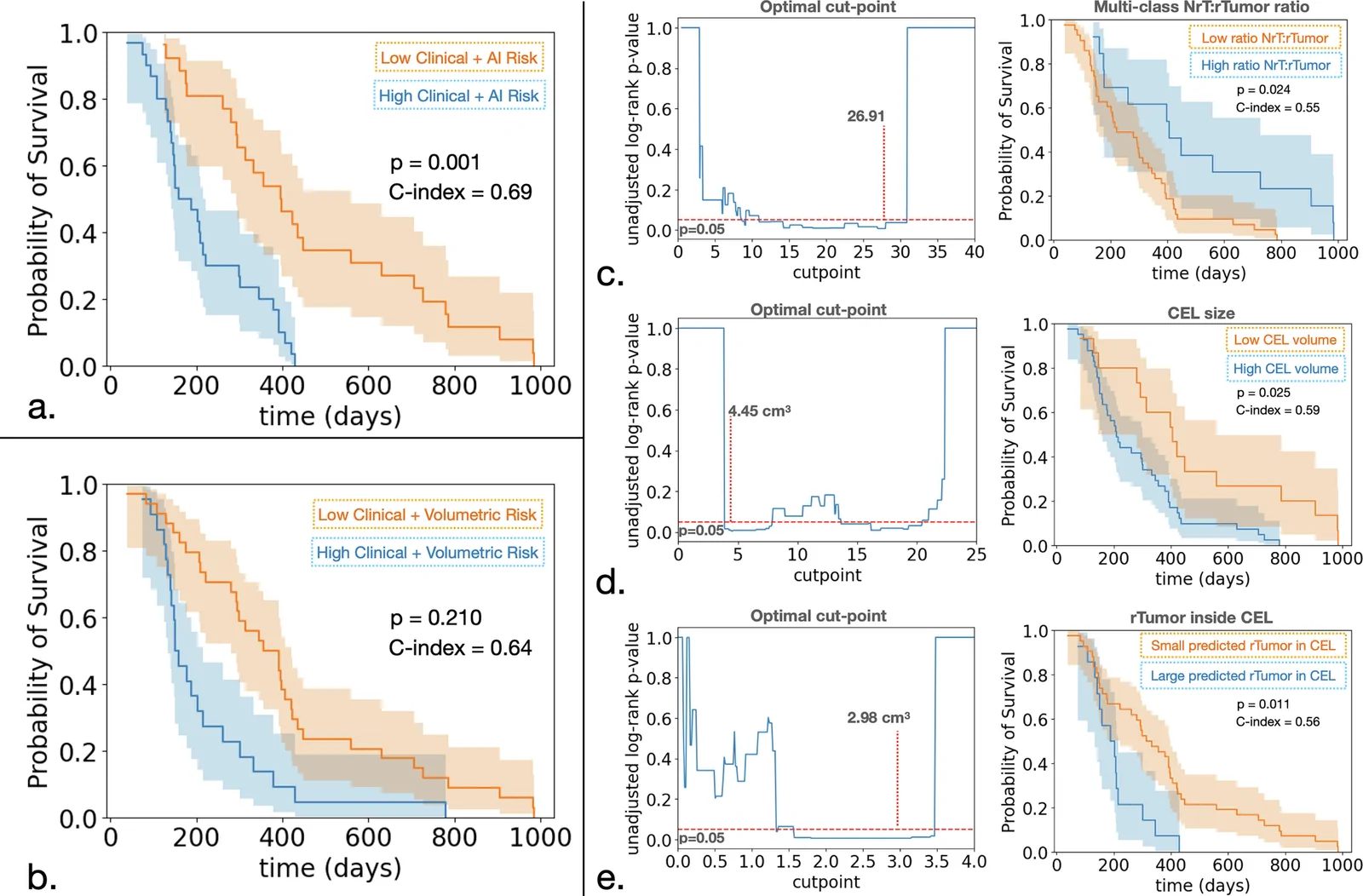
Survival analysis for 56 glioma patients not used for model development. Kaplan–Meier (KM) curves for all patients generated from the predicted partial hazards from the final multivariate elastic Cox Proportional Hazards (CoxPH) model (**a**) and the model using only volumetrics and clinical features (**b**). Features were scaled for models a and b. Cut-points were determined using the unadjusted maximally selected rank statistic. *P* value, and C-Index are obtained from the multivariate elastic CoxPH model fit with lifelines version 0.30.0. KM curve and cut-point analysis for **c** predicted non-recurrent tumor (NrT) ratio to recurrent tumor (rTumor) inside the lesion, **d** contrast-enhancing lesion (CEL) size, and **e** predicted rTumor volume inside the CEL are shown. *P* values and C-Indexes are obtained through fitting a univariate CoxPH model to each of the features.

For the top performing models, RF and soft voting ensemble, T1 post-contrast (T1c), DSC PH, and Apparent Diffusion Coefficient (ADC) were consistently the top three features for classifying rTumor from NrT. Changes in testing AUROC for all imaging features using an RF are shown in the violin plots in Fig. 4a, and both top-performing models in Supplementary Fig. 5. Decision boundaries when fitting across combinations of these features are displayed in Fig. 3b–d and Supplementary Fig. 5 and consistently show high T1c with high ADC, and high T1c with low DSC PH as indicators of NrT.

### Survival analysis

**Fig. 5:**
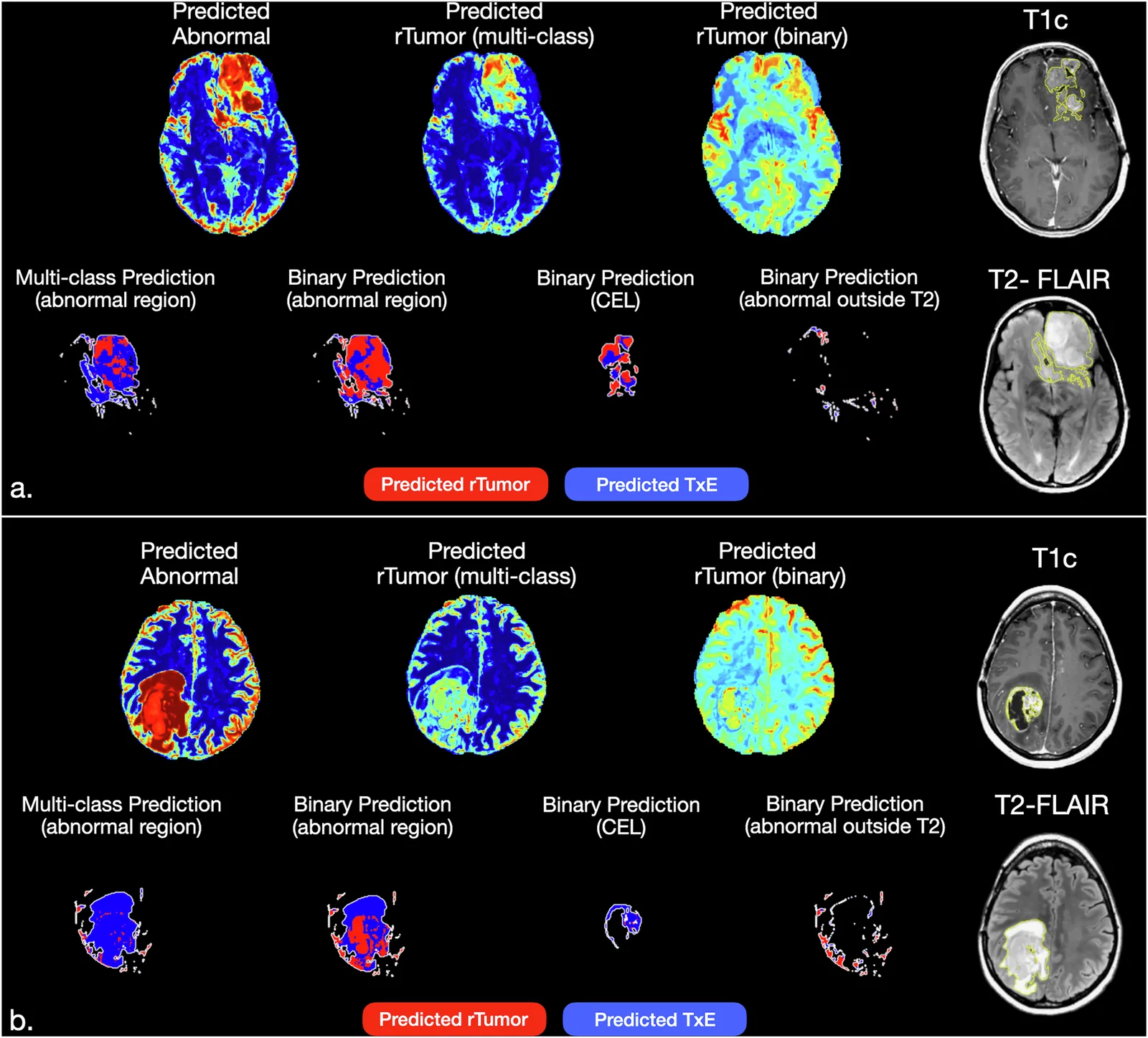
Two examples of spatial maps generated for patients in the survival analysis, with key AI-derived spatial signatures associated with survival. **a** Highest predicted hazards by the most significant CoxPH model, with true survival of 75 days. **b** 9th lowest predicted hazards by the same model with a true survival of 728 days. The low-risk patient has a greater ratio of predicted non-recurrent Tumor (NrT) to recurrent tumor (rTumor) and a relatively small amount of predicted rTumor in the contrast-enhancing lesion (CEL) compared to the high-risk patient. Patients were chosen at each end of the risk spectrum with larger CEL sizes to illustrate the differences in the AI-derived spatial features.

The final features for the elastic Cox proportional hazards (CoxPH) model, using only clinical and traditional volumetrics, as well as the model incorporating AI-derived features, are shown in Table 3. The elastic CoxPH model using all features had a Harrell’s concordance index (C-Index) of 0.69 and *p* = 0.0005 from the likelihood-ratio test during model fitting. The optimal correlation threshold for grouping features was 0.85, as shown in Supplementary Fig. 6, with feature stability illustrated in Supplementary Fig. 7. Many of the artificial intelligence (AI)-derived features were stable across grouping thresholds, most notably the volume of predicted recurrent tumor inside the CEL and the multi-class ratio or NrT to rTumor inside the predicted abnormal area. When loosening the grouping constraint, including more AI-features resulted in a stronger association with survival. Modifying the correlation threshold did not affect the final features for the model that only included clinical and standard volumetric features, which had a C-Index of only 0.644 that did not reach statistical significance (*p* = 0.21). Kaplan–Meier (KM) curves using predicted partial hazards for both models and optimal cut-point analysis are shown in Fig. 4 for the significant volumetric measurements in univariate analysis: size of the CEL, size of the predicted rTumor within the CEL, and the ratio of predicted NrT to rTumor in the lesion obtained from the multi-class model predictions. Hazard ratios, reported as percent of risk increase per 1 standard deviation increase, and *p* values for each metric in both multivariate elastic CoxPH models are listed in Table 3. A higher predicted rTumor in the CEL was more significantly associated with worse survival (*p* = 0.01) than CEL size (*p* = 0.03), whereas a higher predicted NrT to rTumor ratio was determined to be significantly protective (*p* = 0.02). Figure 5 highlights 2 example cases of high and low partial hazards derived from the best model, along with spatial maps and key AI-derived spatial signatures of risk.

**Table 3:** Multivariate elastic CoxPH models using AI-derived metrics from spatial maps

Volumetrics + clinical features	Hazard ratio^a^	*P* value
*Age*	1.229	0.195
*Predicted IDH mutation status*	0.960	0.811
*Gross Total Resection (GTR) status*	0.783	0.159
*Sex*	0.923	0.602
*Near GTR status*	1.007	0.965
*CEL size*	1.305	0.110

AI + volumetrics + clinical features		
*T2L* + *CEL size*	1.326	0.045
*NrT prediction uncertainty (manual lesion)*	4.865	<0.0005
*NrT prediction uncertainty (abnormal)*	0.207	<0.0005
*rTumor prediction uncertainty*	0.793	0.098
*Age*	1.448	0.028
*Multi-class ratio of NrT to rTumor*	0.625	0.016

^a^Hazard ratios of volumetric measurements are reported per 1 standard deviation increase.

## Discussion

By leveraging our unique dataset of pathologically confirmed tissue samples with known coordinates on pre-surgical, multi-parametric anatomical and physiological MRI, we were able to successfully spatially map areas of NrT and rTumor in lesions from patients with HGG at the time of suspected recurrence, avoiding complications of tissue heterogeneity that confound most lesion-level analyses and prediction models. The proportion of NrT to rTumor within the lesion generated by our models was more significantly associated with overall survival compared to the conventional CEL volume and/or known clinical measures, further demonstrating clinical utility.

From a methodological standpoint, this study addressed several practical limitations present throughout the literature for distinguishing treatment-related effects and non-recurrent tumor components of the lesion in imaging from true tumor recurrence. Because most prior studies attempt to classify the entire lesion as rTumor or TxE, they do not provide spatial information, despite high patient-level performance. Our model performance supports the feasibility of spatial classification, while also highlighting the inherent problem with whole lesion classification, as the majority of patients experience a mix of these phenomena^33^. Preserving spatial information on tumor heterogeneity could provide information on the most likely location of tumor recurrence and where abnormal imaging is apt to reflect non-recurrent tumor and the effects of treatment. This information has the potential to guide where tissue should be sampled during surgery to maximize accurate histopathological diagnosis, as well as potentially improving the effectiveness of surgical intervention and reducing unnecessary risk to the patient. The spatial maps can also be generated at any time point to further track how subsequent treatment influences the presence of rTumor within contrast enhancement or T2-hyperintense signal on conventional MRI.

Our best-performing model achieved a test AUROC of 0.74 ± 0.08 when ADC, Fractional Anisotropy (FA), PH, and %Recov were included in the model along with anatomical MRI, various machine learning models were ensembled, the data were stratified to balance the proportion of NrT samples within each round of validation and testing, and a repetitive testing strategy was applied. Consequently, this approach yielded a more stable, generalizable result compared to other studies, which often either lacked ground truth histopathology, utilized a relatively small number of samples, or validated models on a single test set. With a larger number of samples and repetitive testing with no data leakage, we demonstrated a level of robustness beyond what has been previously published^20–28^.

The models showed high T1c with high ADC and high T1 with low DSC PH as indicators of NrT, which is consistent with the current literature^15–18^^,^^34,35^. T1c enhancement is the hallmark signal of TxE, as T1 enhancement can be the result of vasodilation and BBB disruption due to treatment which mimics the appearance of tumor recurrence and thus is limited in its ability to distinguish rTumor for TxE on its own, however it was found to be important when used in conjunction with imaging parameters from diffusion (ADC) and perfusion that measure restricted diffusion in the presence of infiltrative tumor^35^ and hypervascularity^15–18^ respectively. In our dataset, T1c was significantly positively associated with NrT (*r* = 0.25, *p* < 0.0001). This could potentially be explained by the presence of enhancing and non-enhancing rTumor samples with different imaging signatures^19^, and rTumor samples having more areas of non-enhancing infiltration or pseudo-response^36^. Low or no T1 enhancement indicates the lack of blood-brain-barrier breakdown, in combination with high perfusion (DSC PH) could point to vascular recruitment in the presence of infiltrative tumor, which is consistent with our findings. Including rCBV did not improve performance when added in conjunction with DSC PH. One possible explanation for this is that the rCBV was significantly strongly associated with DSC PH (Pearson’s *r* = 0.79, *p* < 0.00001), which may have caused redundancy and decreased model performance due to multicollinearity. Although rCBV has been previously reported as a biomarker for distinguishing rTumor from pseudoprogression and the effects of treatment, we found that including rCBV in place of DSC PH resulted in a significant reduction of performance. This could be explained by the fact that DSC PH was slightly more negatively associated with NrT than rCBV across the dataset (*r* = −0.17, *p* = 0.006 vs *r* = −0.15, *p* = 0.014) and that rCBV was significantly negatively associated with DSC %Recov (*r* = −0.19, *p* = 0.002), whereas DSC PH showed no significant correlation (*r* = −0.05, *p* = 0.44), indicating that DSC PH and %Recov together provided the most unique and complimentary information, improving model performance. Since rCBV values were obtained with leakage correction^37^, their values may be reduced compared to nPH values in locations where vessels are leaky and subsequently where %Recov is low, decreasing the magnitude of its effect in comparison to PH, which does not correct for leakage. While rCBV has been shown to be more strongly associated with tumor recurrence on its own, in context with %Recov, it may capture some overlapping information, which potentially reduces the model’s ability to jointly exploit different measures of leakage and hypervascularity.

Ensemble uncertainty metrics were significantly correlated to prediction failures for rTumor. Epistemic uncertainty provides confidence that samples are within the training distribution, demonstrating the reliability of predictions for rTumor. This was not necessarily the case for predictions of NrT, which showed increasing trends in epistemic uncertainty that were correlated with prediction success. This could be due to the fewer number of NrT samples, reducing the model’s ability to assess out-of-distribution samples; however, this does support the model’s ability to associate imaging anomalies with NrT.

The predicted ratio of NrT to rTumor obtained from the generated spatial maps of recurrent tumor were significantly associated with survival, with higher ratios being protective. This further verified that the machine learning model captured global effects of treatment and the lack of concentrated rTumor, indicative of better survival outcomes. The volume of predicted rTumor in the CEL was more significantly associated with survival than the volume of contrast enhancement, indicating enhanced sensitivity of the AI-derived measure to tumor recurrence. These results further validate our approach and support the potential of these AI-derived spatial signatures as noninvasive imaging biomarkers of recurrent tumors or as additional prognostic factors. Predicted IDH wild-type as a risk factor was insignificant in the multivariate clinical and volumetric CoxPH model, with an HR of 0.960 (*p* = 0.811). This could be explained either by inaccuracies of the IDH status prediction or because CEL size was significantly correlated to IDH wild-type (*r* = 0.33, *p* = 0.01) and more strongly associated with survival in this cohort, given the small amount of predicted IDH mutants (9/56).

There are also a few inherent methodological limitations. As our models were trained and validated on data from a single institution, we were not able to assess the generalizability across institutions as part of this initial study. However, the lengthy duration of this study naturally resulted in some heterogeneity in sequence acquisition parameters that may have resulted in a broader imaging distribution that potentially could have negatively contributed to the modest performance of our models, but may also improve future model generalizability to unseen data acquired at other sites. By utilizing varying acquisition parameters, it is likely that our estimate of model performance more closely resembles that of what is observed in clinical practice, where imaging distributions can shift both over time and due to variations in sequence parameters across scanner platforms. Although our standardization protocol using normal-appearing brain attempted to mitigate this variability, future studies evaluating the effects of varied imaging protocols on model performance and generalizability are necessary.

Although the vast majority of our tissue samples were acquired with a biopsy needle before opening the dura and resecting the tumor tissue, where most reported shifts have been 2–5 mm with maximum shifts of <10 mm, it is highly likely that this shift is the main contributor to reducing the accuracy of our results, despite our efforts to minimize this error by using 10 mm cubic patches as inputs to our models, performing extensive quality control through manual visualization of each tissue sample location, and excluding any samples that were structurally inaccurate. While 10 mm patches were used to mitigate tissue shift, there remains an inherent trade off with including imaging signatures of mixed tissue types in the ROI, including pure treatment-induced effects, recurrent tumor, necrosis, cerebrospinal fluid (CSF), cavity, cytotoxic edema, and normal-appearing tissue, which may have reduced performance due to the contribution of these tissue types to the mean values for each imaging modality.

Other limitations arise specific to our survival analyses. Key metrics previously established to be associated with survival—such as the T2 hyperintense and contrast-enhancing volumes of residual tumor^38^ and the true IDH mutation and O^6^-methylguanine-methyltransferase (MGMT) methylation status^39^—were not available for this analysis and may have strengthened the model’s association with survival. Understanding how this molecular information may influence the link between predictions of rTumor to survival outcomes may provide additional clinical utility and would be a valuable direction for future study. Although less of a concern, fully automated lesion segmentation for tumors post-treatment is still an ongoing challenge, and our manually defined lesion volumes may have suffered from small inaccuracies despite neuro-radiologist review of difficult cases, which could have potentially impacted our survival analysis. Finally, while previous studies primarily focus on the identification of TxE, our designation of NrT comprised a broader definition of non-tumoral lesion compartments, which may not be applicable to answering clinical questions requiring specific identification of treatment-induced changes.

In conclusion, our results demonstrate a step towards clinical feasibility for noninvasively distinguishing between rTumor and NrT, while using a tiered approach for first classifying normal appearing brain and estimating model uncertainty. The association of the AI-derived metrics with survival further validates our model. The proposed spatial maps could provide additional pieces of information to clinicians when reassessing prognosis, surgical planning, and tissue sampling, as well as determining the appropriate course of treatment for glioma at the time of suspected recurrence.

## Methods

### Patient characteristics

Institutional Review Board approval was obtained at our institution to prospectively enroll consecutive patients with a 2016 World Health Organization (WHO) pathology-confirmed diagnosis of diffuse glioma who underwent surgery for a suspected recurrence between 2007 and 2018. A total of 135 patients (median age, 49; range, 19–77) were enrolled who provided written informed consent to participate and underwent MR imaging between 1 and 3 days prior to surgical resection and tissue sample collection, with the exception of 5 patients who had surgery 4–7 days after imaging. Suspected recurrence was defined by evolving criteria that included but were not limited to clinical deterioration, conventional increases in size of the existing lesion on anatomical imaging (RANO 1.0/2,0)^12,40^, the presence of a new lesion, or changes on advanced imaging (i.e., using perfusion- or diffusion-weighted MRI and/or Fluoroethyl-L-Tyrosine (FET)-PET). Demographics were typical for this population, as shown in Table 4. Although the majority of patients received an initial surgical resection followed by the standard-of-care radiotherapy plus temozolomide, treatment history varied among patients, and 15 patients were clinically diagnosed with TxE lesions. From each patient, 1–4 tissue samples were obtained (mean samples/patient was 1.88, with 1.78 samples/patient originating from the CEL and 1.63 samples/patient from the non-enhancing lesion (NEL)). A tissue sample was only included in the analysis if it: (i) had a conclusive histopathological outcome; (ii) had quantifiable anatomic imaging data; and (iii) overlapped with the CEL or NEL after visual verification of the accuracy of the coordinates on anatomical images using 3-plane screenshots taken during the surgery. This resulted in 254 tissue samples with visually verified spatial coordinates on imaging that could be used in our analysis out of an original 343 acquired tissue samples. All patients gave informed consent to participate in research according to guidelines established by the Institutional Review Board (IRB) of the University of California, San Francisco (protocol #10-03588), in accordance with the Declaration of Helsinki.

**Table 4:** Patient demographics for model development and survival analysis

Model development				
	Sex	Grade	Histological Type	Count
*Male*	53% (71)			
*Female*	47% (64)			
*Grade IV*		32% (43)		
*Grade III*		36% (48)		
*Grade II*		20% (28)		
*Grade I*		1% (1)		
*Treatment Effect*		11% (15)		
*Glioblastoma*			29% (39)	
*Oligodendroglioma*			27% (37)	
*Astrocytoma*			23% (31)	
*Other*			310% (134)	
**Survival analysis**				
*Male*	66% (37)			
*Female*	34% (19)			
*Sub-total Resection*				57% (32)
*Gross Total Resection (GTR)*				23% (13)
*Near GTR*				20% (11)
*Predicted IDH wild-type*				84% (47)
*Predicted IDH mutant*				16% (9)

A separate dataset of 56 patients (median age, 54; range, 23–84) with GBM as determined by WHO 2016 criteria (Table 4) and acquired using the same MRI pre-surgery protocol acquired prior to surgical resection and tissue sample collection, but was either lacking coordinates of the tissue on imaging or did not pass the initial inclusion criteria for tissue samples, was used to further validate the clinical utility of our models by associating parameters derived from our predicted spatial maps with overall survival.

### Tissue sampling and histopathological analysis

A minimum of 4 tissue samples at least 1 cm apart were preoperatively planned to maximize heterogeneity of imaging features within the T2 lesion. Tissue sampling and planning utilized an intraoperative navigation system (BrainLab or Surgical Stealth) that displayed both the post-contrast T1-weighted and T2-Fluid Attenuated Inversion Recovery (FLAIR)images. Targets for tissue sampling were selected from both the contrast-enhancing and non-enhancing components of the lesion based on a variety of imaging characteristics from metabolic and physiologic imaging, including the most metabolically active and cellular regions based on choline to N-acetylaspartate (NAA) index (CNI), and low ADC levels and hypoxic regions denoted by the presence of lactate and elevated rCBV. This strategy enabled the acquisition of samples from multiple regions within the same lesion that represented areas of heterogeneous tumor biology as defined by the histopathological tumor score, cellularity, proliferation, hypoxia, and presence of abnormal blood vessels, while simultaneously avoiding edges of cavities and necrotic regions and considering the accessibility of the tissue target to the surgeon.

During surgery but prior to tumor resection, samples were obtained from the targeted locations using a 2 mm biopsy needle before opening the dura, and the surgical navigation system recorded the actual coordinates on MRI from where each sample was obtained, along with screenshots of the needle tip overlaid on the MRI in 3-planes to allow for subsequent visual confirmation of the location on the pre-surgical MRI using in house software. Samples were immediately formalin fixed and subsequently paraffin-embedded.

Hematoxylin and eosin stained slides from tissue samples were evaluated by a board-certified pathologist (J.J.P.) and assessed for the presence of tumor cells, necrosis, and treatment-related abnormal vasculature. The presence of tumor cells was scored as follows: 0 = no tumor present; 1 = less than 10% infiltrating tumor with rare cells, 2 = infiltrating cellular tumor, and 3 = highly cellular infiltrating tumor involving >75% of the tissue. Samples that had tumor scores of 2 or 3 were considered recurrent tumors, while tumor scores of 0 or 1 comprised the non-recurrent tumor (NrT) class. These latter samples exhibited either pathological characteristics associated with known treatment-induced changes, such as hyalinized blood vessels, gliosis, necrosis, etc., or were defined as pathologically normal within the lesion. This resulted in 179 tissue samples being identified as rTumor, while 75 were designated as the NrT class.

In addition to regions of pathology, 1cc normal-appearing brain regions were selected on imaging for model development. The selection of normal appearing brain included contralateral regions of white matter, gray matter, and CSF, with at most three regions selected randomly for each patient, totaling 395 normal appearing tissue regions. The three cubes were manually selected per patient using in-house software to obtain signatures representing each tissue compartment. To avoid significant partial voluming, at least 60% of the selected cube was within the designated anatomical region.

### Image acquisition

Multi-parametric MR images (mpMRI) using a protocol consisting of anatomical, diffusion-weighted, and perfusion-weighted imaging were collected from all patients suspected of recurrence prior to surgery on a 3 Tesla (3 T) scanner (GE Healthcare) with an 8-channel head coil. 3D T1c, T2-FLAIR, and Diffusion-weighted (*b* = 1000 s/mm^2^ and either 6 gradient directions and 4 excitations or 24 gradient directions and 1 excitation, or *b* = 2000 s/mm^2^ and 55 gradient directions, with repetition time (TR)/echo time (TE) = 1000/108 ms and voxel size = 1.7–2.0 × 1.7–2.0 × 2.0–3.0 mm) images were acquired as part of the clinical pre-surgical MRI exam. DSC perfusion-weighted images (TR/TE/flip-angle = 1250–1500/35–54 ms/30°–35°) were obtained before, during, and following a 3 mL/s bolus injection of 0.1 mmol/kg body weight gadolinium diethyltriamine pentaacetic acid using a series of T2*-weighted echo-planar images (128 × 128 matrix, slice thickness = 3–5 mm, 7–24 slices with 60–80 time points). DSC was performed without pre-loading and using a low flip angle to ensure similar post-bolus signal quantification, which would affect the %Recov measurement.

### Image processing and alignment

A pipeline that utilized components of FMRIB’s Diffusion Toolkit was applied to estimate relevant ADC and FA maps from the diffusion-weighted imaging and diffusion tensor imaging data as previously described^41^. From the DSC perfusion data, non-parametric PH and %Recov were quantified using in-house software^42^. rCBV maps were generated using the bi-directional, leakage-corrected perfusion MRI signal^37^. After alignment of anatomic, diffusion, and perfusion data to the T1 post-contrast image using either FMRIB’s software library (FSL) Linear Image Registration Tool^43^ or Slicer’s BRAINSFit tool^44^ with B-spline warping, each image contrast and quantitative map was interpolated to 1 mm^3^.

### Lesion and brain mask generation for normalization and survival analyses

T2- and CEL masks were either previously defined as part of prior studies^19,45^ if used in defining normal-appearing brain regions for parameter normalization during training, or, in the case of the survival analysis cohort, were obtained via combinations of semi-automated approaches^46,47^ followed by manual editing by TLL (an expert senior scientist with over 15 years of experience performing this task). Any cases that were identified as potentially challenging were additionally verified by our study neuro-radiologist (JEVM) and further adjusted if appropriate. To generate the mask of normal-appearing brain tissue required for our standardization procedure, brain extraction was first performed on the T1c image by either FSL’s Brain Extraction Toolkit (BET)^48^ or HD-BET software^49^, before subtracting a total lesion mask (the sum of the CEL and T2L masks). To account for differences in acquisition parameters over the 12-year study duration and prevent overfitting, the mode and standard deviation of values within the normal-appearing brain mask were used to standardize all images and maps while preserving the relationship between normal-appearing brain and lesion.

### Input patch generation

For each tissue sample acquired, cubic 10-mm^3^ ROIs centered at the location of the spatial coordinates recorded during surgery were generated. This size was selected to balance the need to restrict the ROI to the vicinity of the sampled tissue and account for any potential errors introduced by tissue shift, which has been previously reported as between 2 and 5 mm (with maximum shifts of <10 mm)^50,51^. All patches were then visually verified for correctness on anatomical imaging using in-house software and screenshots taken during the surgery. Any sample that either had a mismatch between the coordinate locations and visualized location on imaging or contained mostly necrosis or resection cavity due to the tissue being at the boundary of these regions was excluded. The spatial mean across each of the resulting *N* cubic patches surrounding the center of the tissue sample coordinates was stacked from each normalized image contrast or quantified map, resulting in an [*N* × *C*] matrix used as input to the machine learning (ML) models.

### Classification of recurrent tumor

**Fig. 6:**
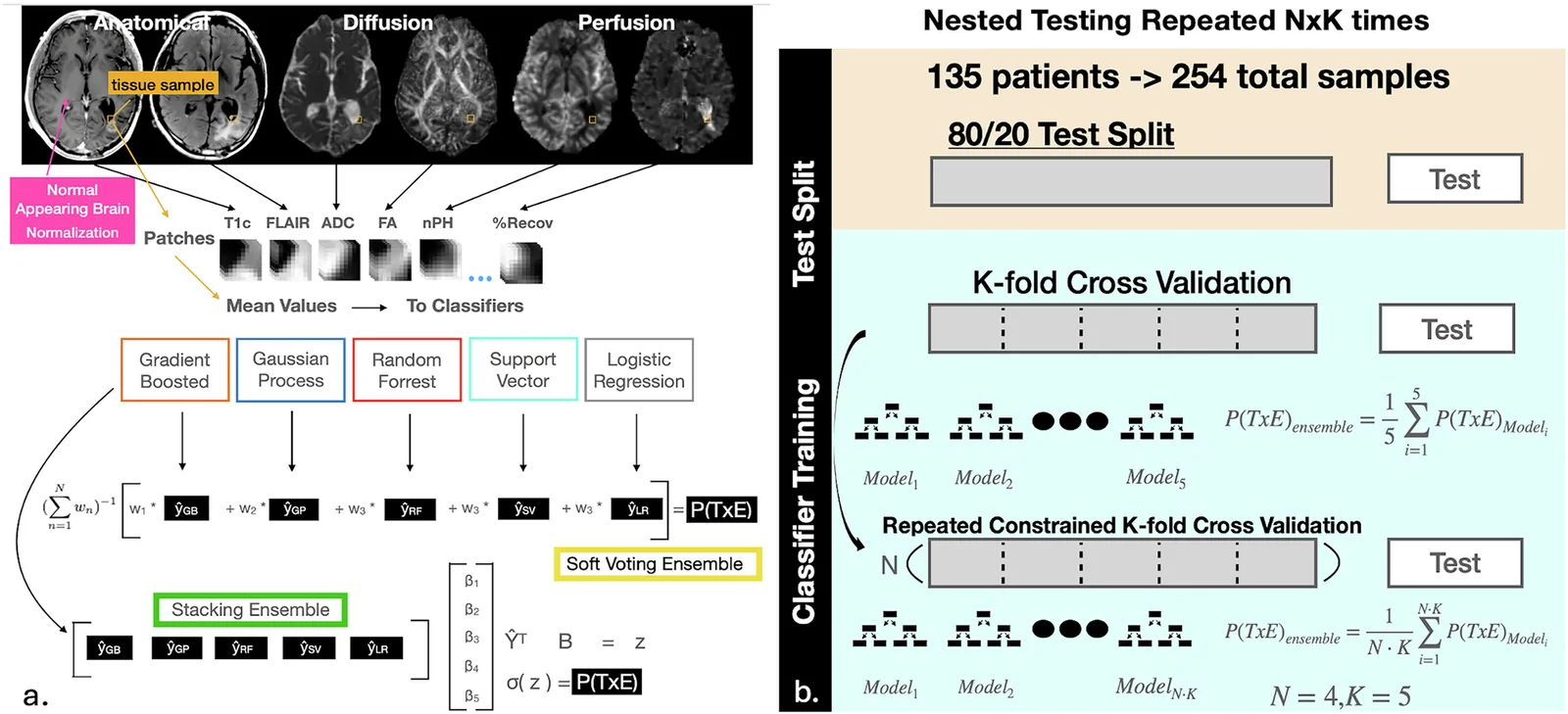
Overview of model development. **a** Multi-parametric MRI data and modeling procedure. Pre-surgical image patches surrounding known coordinates of histopathologically evaluated tissue samples were used as inputs to the machine learning models. **b** Models were trained and compared using nested testing with Repeated Constrained k-fold Cross Validation (RCCV) using tight constraints on patient grouping but loose constraints on class proportions with folds *K* = 5, and repetitions *N* = 4 to generate 20 ensembles of 20 models which were each used to average predictions on each of the 20 seperate test sets.

For binary classification of rTumor and NrT, SVM, RF, GB, LR, GP, a soft voting ensemble, and a stacked regression model were implemented, as shown in Fig. 6. For multi-class classification that included the addition of normal-appearing brain samples, the best singular model, RF, was used due to the memory constraints for ensembling across model types and datasets.

### Data splitting and model training

To generate an ensemble of models across unique subsets of the data, we implemented a Repeated Constrained k-fold Cross Validation (RCCV) approach, which extends stratified group k-fold cross-validation. RCCV enforces tight patient grouping constraints to prevent data leakage and tunable constraints on stratification to allow for greater variation across subsets in repetitions, while maintaining uniqueness and large validation set sizes. Samples were split by patient into *K* = 5 splits, constraining the proportion of the minority class (NrT) in each split between ±10% from the population proportion for the binary task and ±1% for the multi-class task to account for the abundance of normal appearing brain samples. This nested splitting strategy was repeated *N* = 4 times to yield 20 unique subsets of training and validation data for a given test set (Fig. 6), with no overlapping of patients between training and testing. Each sample was validated *N* times, facilitating an estimate of ensemble performance when the predictions were averaged. This process allowed us to achieve more stable performance metrics without limiting the total number of models generated. For binary classification, the synthetic minority oversampling technique (SMOTE)^52^ was used to oversample NrT in each training fold to equal proportions as the majority class (rTumor), while in the multi-class setup, all classes were oversampled to equal proportions of the normal-appearing brain class for training. When initially splitting off the test sets for repetitions of testing, a small remainder of patients in each training set was moved to testing to ensure divisibility by 5 in the training set to facilitate even splitting while maintaining uniqueness for each test set of 30 patients. Hyper-parameter tuning used grid-search on the first inner loop of training and validation. For comparison, to further demonstrate the validity of our RCCV approach, we also created five unique test sets with grouped and stratified cross-validation. For this set of experiments, five independent tuning rounds were performed, one for each train/test split, to determine the optimal set of hyperparameters separately for each round of testing. RCCV was used on the training sets for each corresponding test set to generate the ensemble of models.

### Model evaluation and failure characterization

Models were evaluated using the AUROC as the performance measure. For the best-performing models, Youden’s J-index was used to obtain thresholds during validation, with the optimal tradeoff between specificity and sensitivity determined from each ensemble. Multi-class predictions were optimized using Youden’s J-index in a tiered approach after prediction as abnormal and renormalizing predictions from rTumor and NrT. Predictions from the binary classification ensemble model were also used to obtain measurements of prediction variance and entropic measures of uncertainty. Epistemic uncertainty, often used as a proxy measurement for assessing confidence that a testing sample is within the training distribution^53^, was calculated using the entropic definition^31^ from subtracting the aleatoric from the total uncertainty as described in Eq. 1:

1 $${U}_{{epistemic}}=-\mathop{\sum }\limits_{c=1}^{C}P\left({y}_{c}|{\theta }^{{ens}}\right)\cdot \log \left(P\left({y}_{c}|{\theta }^{{ens}}\right)\right)+\frac{1}{N}\mathop{\sum }\limits_{n=1}^{N}\mathop{\sum }\limits_{c=1}^{C}p\left({y}_{c}|{\theta }_{n}^{{ens}}\right)\cdot \log \left(p\left({y}_{c}|{\theta }_{n}^{{ens}}\right)\right)$$

where $P\left(y_{c}|{\theta }^{{ens}}\right)=\frac{1}{N}{\sum }_{n=1}^{N}p\left({y}_{c}|{\theta }_{n}^{{ens}}\right)$ are the ensemble predictions, *n* is the number of models trained on different subsets of the data, y_c_ are the class labels, and ${\theta }^{{ens}}$ are the ensemble model parameters. The total, aleatoric, and epistemic uncertainty, as well as the prediction variance, were then evaluated for statistically significant differences using Generalized Estimating Equations implemented in Statsmodels 1.1.12 to account for repeated measures when comparing correct predictions and model failures of classifying rTumor and NrT.

### Feature importance analysis

Permutation feature importance implemented in Sci-Kit Learn 1.1.2 was used to determine the most influential imaging features for classifying NrT. The technique quantifies the reliance of the model on each imaging feature by measuring the decrease in model performance upon shuffling each of the input imaging features. An RF fit on combinations of the top three most important imaging features was then used to generate decision boundaries along these features on a sample test set for visualization.

### Spatial mapping of recurrent tumor

Images were divided into eight stacks of patches in the axial plane for parallel voxel-wise inference to generate whole-brain probability maps of recurrent tumor and maps of epistemic uncertainty. T1c and T2 FLAIR image inputs were first N4 bias corrected using Simple Image Toolkit (ITK)^54^. The multi-class model was used to first generate a map of an abnormal appearing brain, which was combined with a composite mask of T2-hyperintense and T1 CELs to generate a target area over which to perform inference. The best binary classification and multi-class models were then applied to predict voxel-wise probabilities of rTumor and uncertainty metrics inside this area.

### Survival analysis

Spatial maps were generated on a separate set of 56 patients with glioma, who were about to undergo surgery for a suspected recurrence and were not used in model development, using ensembles from the round of testing with the highest AUROC. Fifty-five metrics were extracted from the maps, including the predicted total size of the lesion (from different combinations of the abnormal lesion predictions and anatomical volumes), predicted rTumor volume, predicted NrT volume, ratios of predicted NrT to rTumor in various lesion compartments, average probability of NrT, average uncertainty, average variance, and ensemble metrics within predicted NrT and rTumor areas (Supplementary Table 1). These metrics, along with six clinical variables (i.e., age, predicted IDH status^55^, sex, and EOR) and three anatomical volumetrics (T2-lesion volume, CEL volume, and total lesion volume), were *z*-scored normalized, and used in subsequent analyses to predict overall survival. To remove highly correlated features, AI-derived covariates, anatomical volumes, and clinical features were first grouped by Pearson’s correlation. A single representative feature for each grouping with the lowest *p* value was then selected after fitting a univariate CoxPH model. From this set of features, a backward stepwise approach was then employed to build the multivariate elastic CoxPH model (penalization = 1e-5, L1-ratio = 0.5) until 6 total features were included, following the convention for ten events-per-variable^56,57^. The optimal correlation threshold for grouping was determined by evaluating model performance across a range of correlation threshold values. To assess the relationship between AI-derived metrics and survival, this process was repeated using only the clinical features and standard contrast-enhancing, T2-hyperintense, and total lesion volumes to create a second elastic CoxPH model that served as a baseline comparator. KM curves were generated for key metrics that were significantly associated with survival in univariate analysis for interpretability. Cut-points were determined from the unadjusted maximally selected log-rank statistic, assigning no fewer than 20% of the total patients into any one group.

## Supplementary information

41746_2026_2823_MOESM1_ESM

## Data Availability

The imaging dataset used in this study cannot be shared publicly due to patient privacy and ethical restrictions. However, the results generated from this study can be made available from the corresponding author upon reasonable request.

## Code availability

Code will be made available upon reasonable request according to the guidelines established by UCSF and agencies that provided funding for this study, once all studies using the data have been published.
