# Supplementary material for: Spatially identifying regions of tumor recurrence in patients with suspected recurrent glioma using physiologic MRI and machine learning
Source: NPJ Digit Med. 2026 Jun 25;9:795. doi: 10.1038/s41746-026-02823-y (PMC13669375; doi:10.1038/s41746-026-02823-y)
Supplement: Supplementary file 1 [file 41746_2026_2823_MOESM1_ESM.pdf]

Spatially identifying regions of tumor recurrence in patients with suspected recurrent glioma using physiologic MRI and machine learning

Jacob Ellison MS[1,2,3], Nate Tran PhD[1,2,3], Tracy L. Luks PhD[1], Paramjot Singh MS[1], Angela Jakary BS[1], Tiffany Ngan BS[1], Julia Cluceru PhD[1,2], Joanna J. Phillips MD PhD[4,5], Yan Li PhD[1,2,3], Annette M. Molinaro PhD[4], Valentina Pedoia PhD[1,2,3], Anny Shai PhD[4], Devika Nair BS[1], Javier E. Villanueva-Meyer MD[1,2], Mitchel S. Berger MD[4], Shawn Hervey-Jumper MD[4], Manish Aghi MD[4], Susan M. Chang MD[4], Janine M. Lupo PhD[1,2,3]

[1] Department of Radiology and Biomedical Imaging, UCSF, San Francisco, CA, USA
[2] Center for Intelligent Imaging, UCSF, San Francisco, CA, USA
[3] UCSF/UC Berkeley Graduate Program in Bioengineering, San Francisco & Berkeley, CA, USA
[4] Department of Neurological Surgery, University of California San Francisco, San Francisco, CA, USA
[5] Department of Pathology, University of California San Francisco, San Francisco, CA, USA

Supplementary Material

Manuscript Type: Original Research

Correspondence:

Janine Lupo, PhD
Byers Hall, Box 2530
1700 4th St, Suite 303D
San Francisco, CA 94158
janine.lupo@ucsf.edu, +1 415 502-0646

Supplementary Material

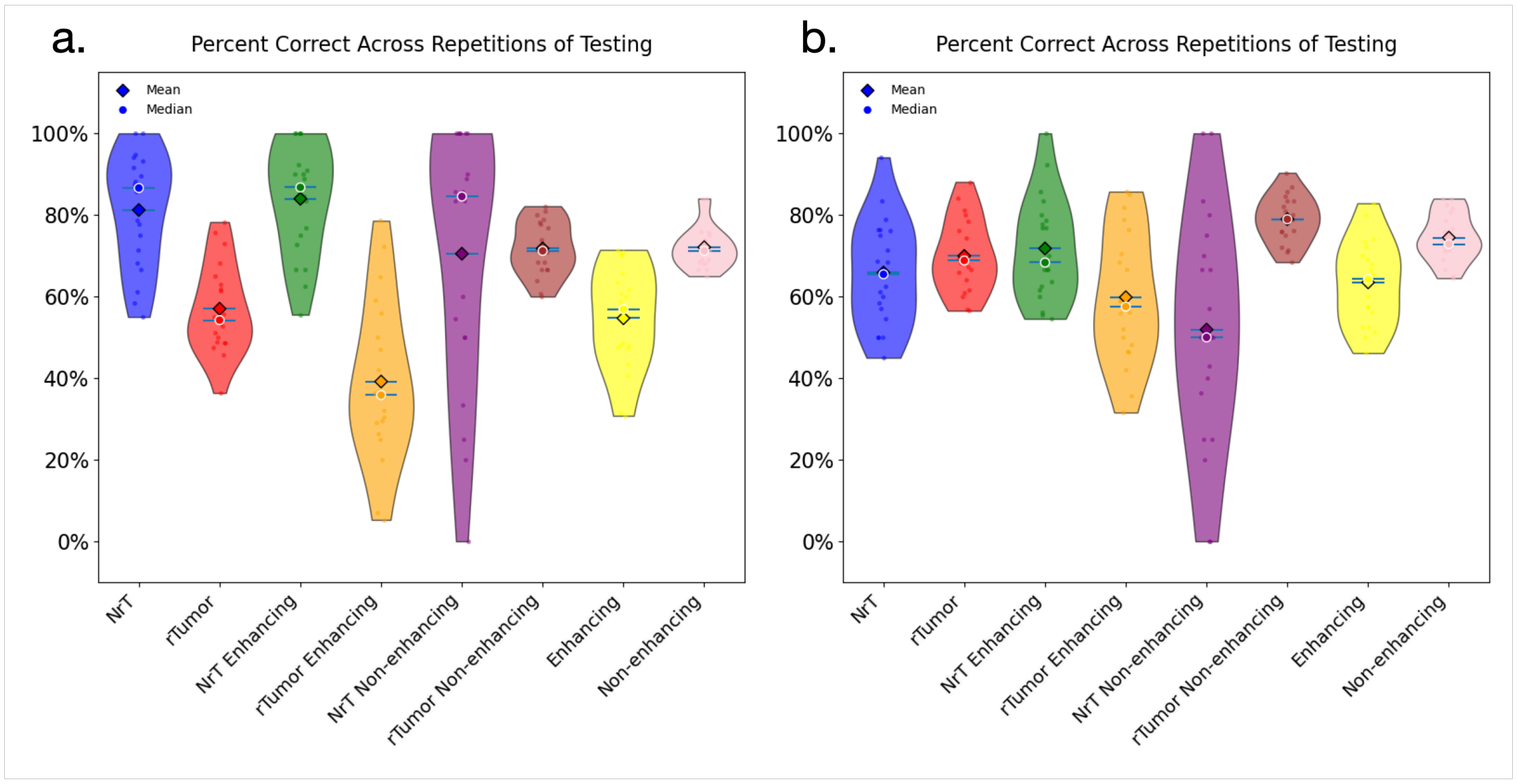

Supplemental Figure 1: Percent correct classification for different groups of enhancing vs non-enhancing samples across repetitions of testing. a) Threshold optimized using Youden's J-Index, b) thresholding prediction probabilities at 0.5. There were 51/24 enhancing/non-enhancing non-recurrent tumor (NrT) samples and 77/102 enhancing/non-enhancing recurrent tumor (rTumor) samples.

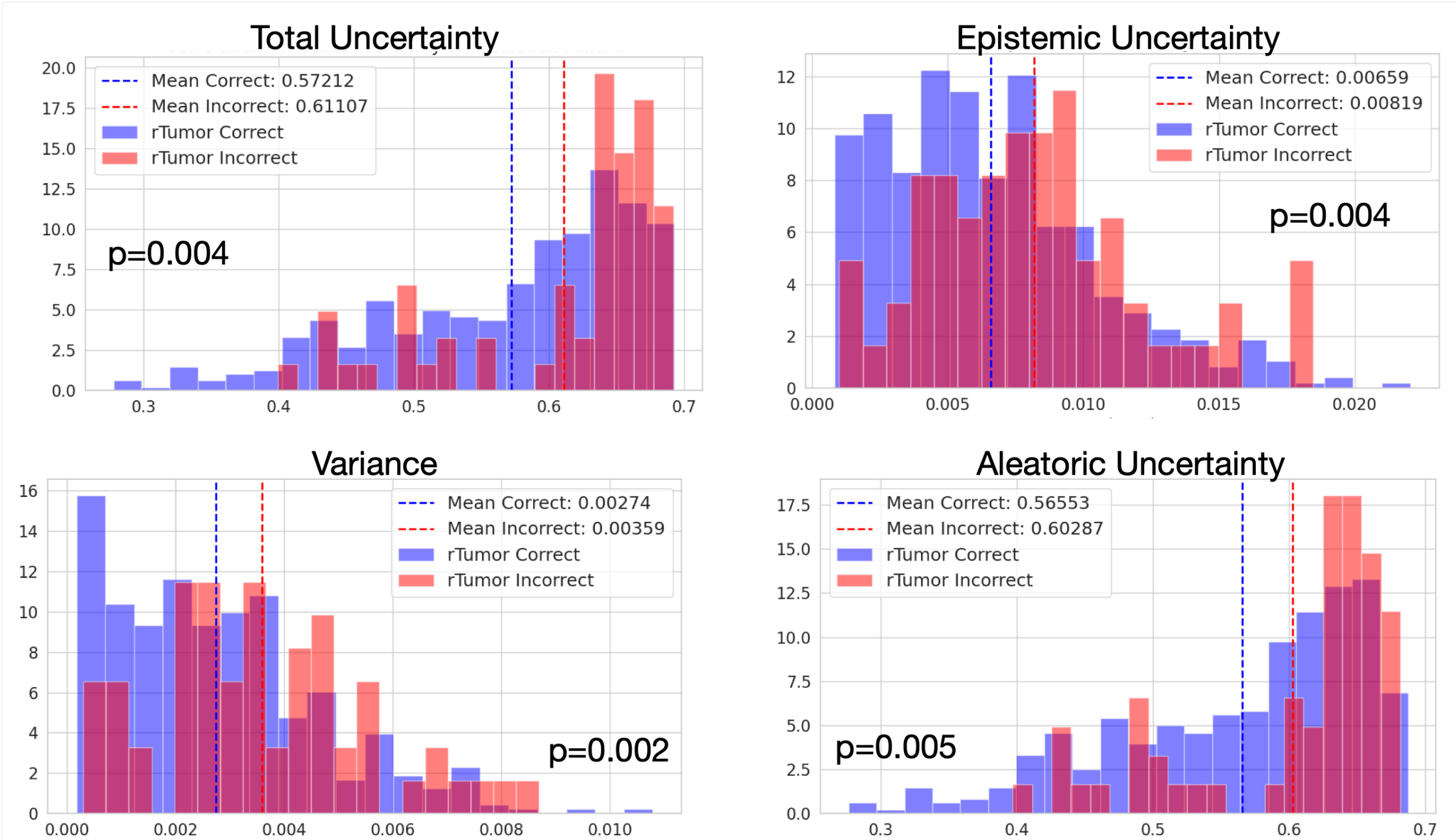

Supplemental Figure 2: Variance, total uncertainty, epistemic uncertainty, and aleatoric uncertainty shown for prediction failures of recurrent tumor (rTumor) across nested repetitions of testing. Generalized Estimating Equations were used to determine significance to account for repeated measures. All uncertainty metrics were significantly correlation to prediction failures of rTumor. Uncertainty metrics were lower with correct predictions of rTumor.

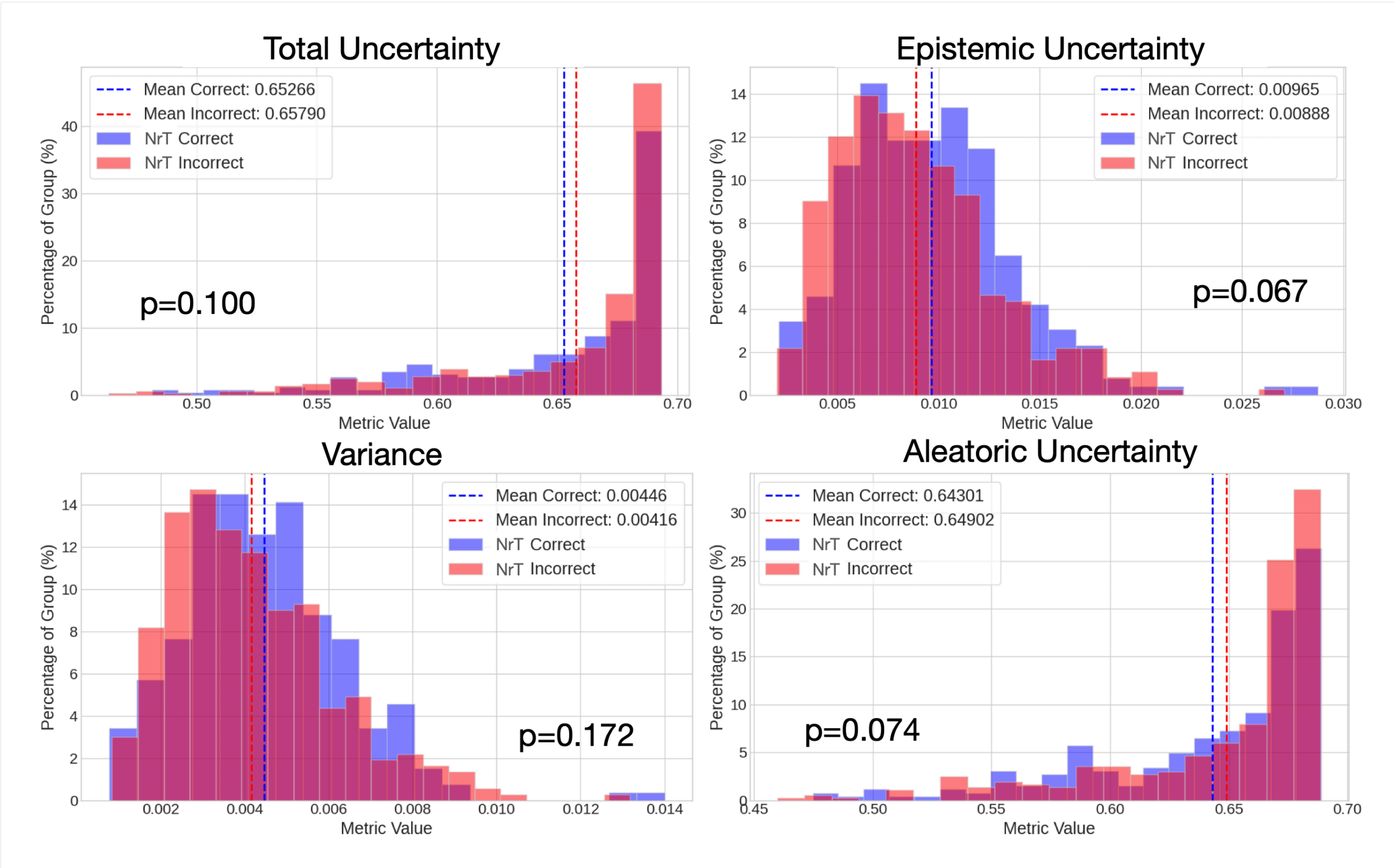

Supplemental Figure 3: Variance, total uncertainty, epistemic uncertainty, and aleatoric uncertainty shown for prediction failures of non-recurrent tumor (NrT) across nested repetitions of testing. Generalized Estimating Equations were used to determine significance to account for repeated measures. Epistemic uncertainty approached significant correlation to prediction failures of NrT. Uncertainty metrics were higher with correct predictions of NrT.

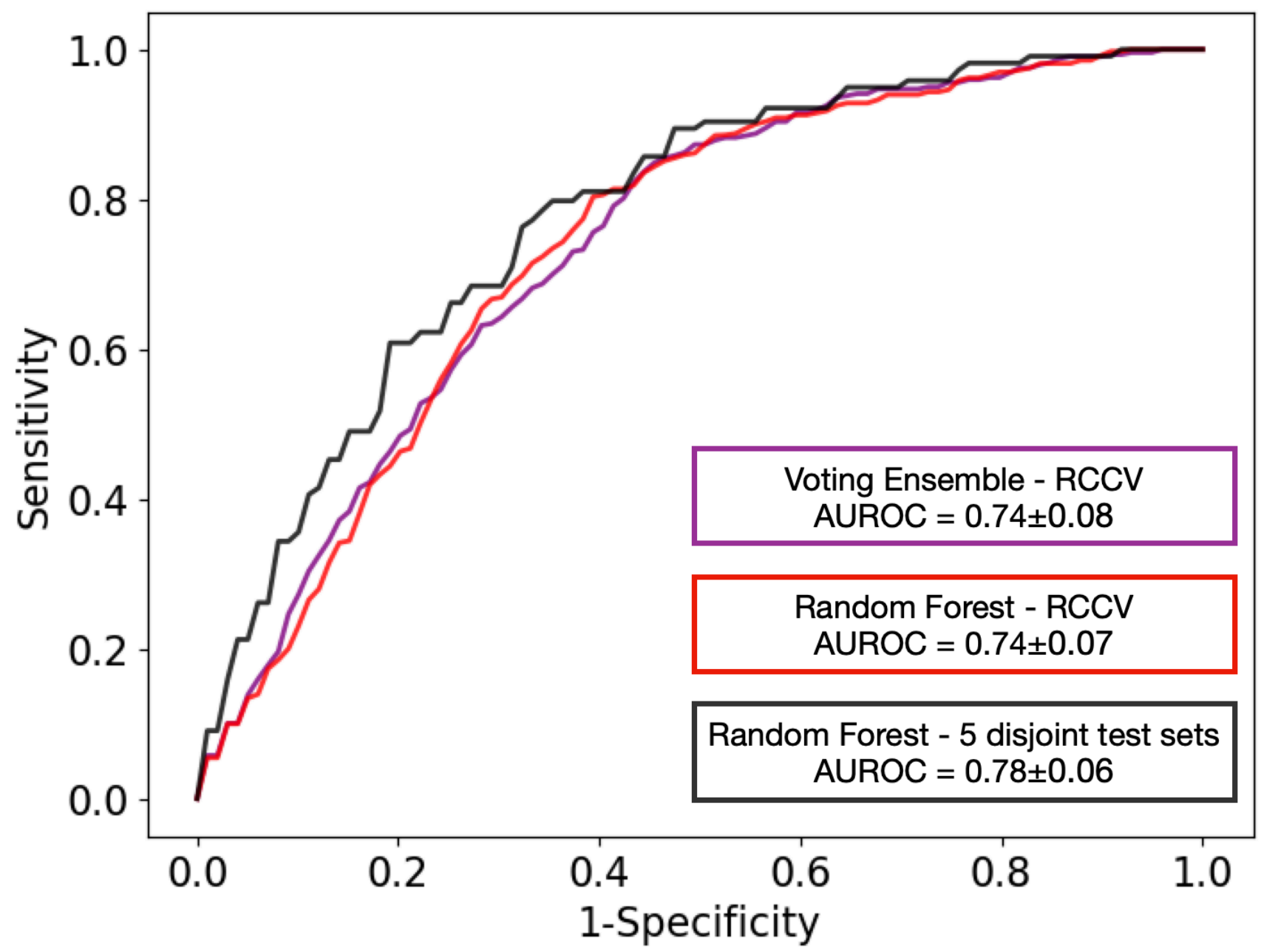

Supplemental Figure 4: Comparison of performance between evaluation protocol using of area under the receiver operating characteristic curve (AUROC) for repeated testing with repeated constrained cross validation (RCCV) and using 5 disjoint test sets that cover all patients without repetition. For the disjoint testing, 5 independent tuning rounds were performed, one for each train/test split. RCCV was used on the training sets to generate an ensemble for each corresponding test set.

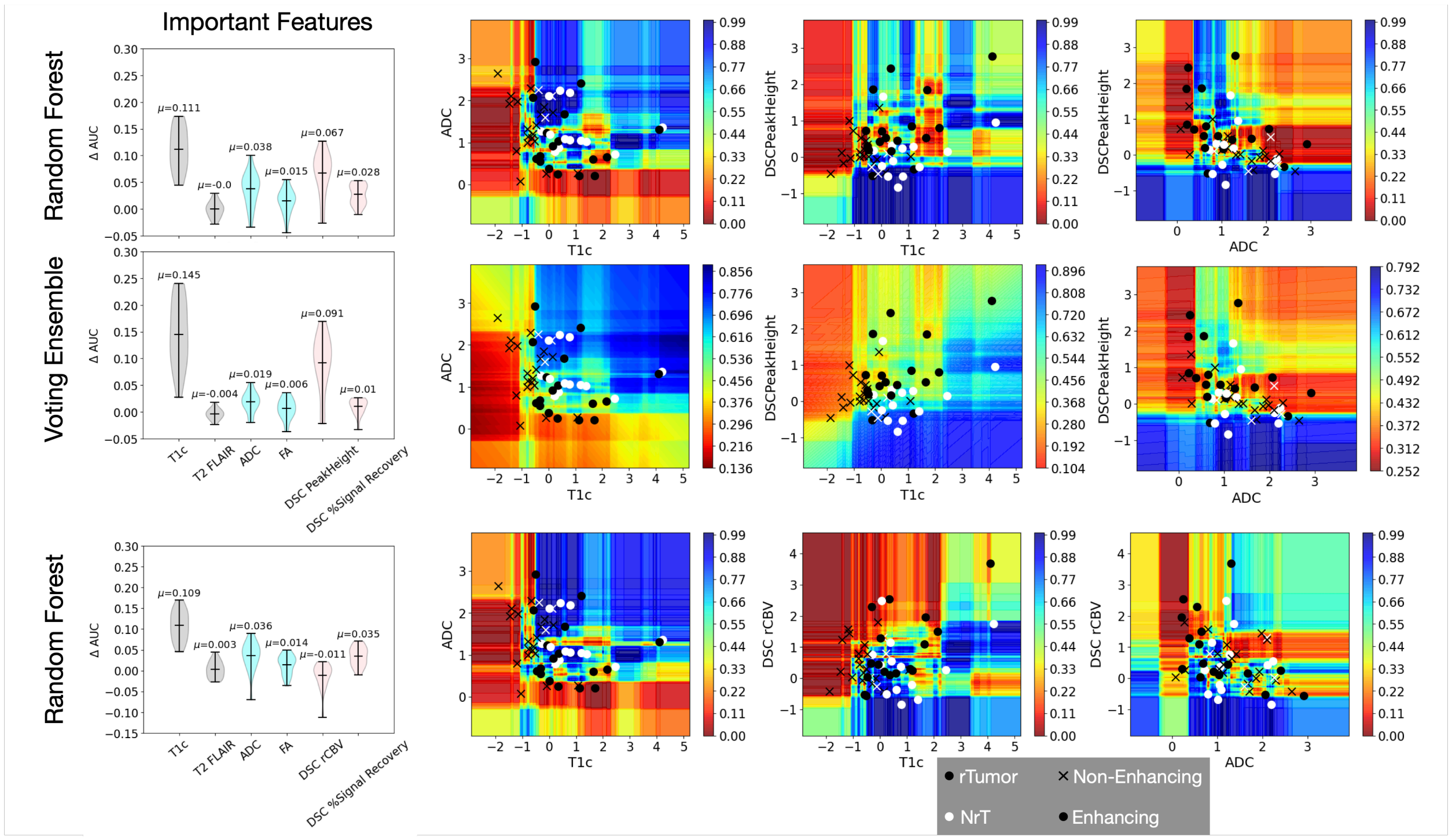

Supplemental Figure 5: Results of permutation feature importance for top two performing models, Random Forest (RF) and soft voting ensemble, and a RF fit to relative Cerebral Blood Volume in place of Dynamic Susceptibility Contrast (DSC) Peak Height. Decision boundaries are shown for the models fit along combinations of the top three impactful imaging features. High T1 gadolinium contrast enhancement (T1c) with high Apparent Diffusion Coefficient (ADC), and high T1c with low DSC Peak Height are consistently indicative of non-recurrent Tumor (NrT). Abbreviations: recurrent tumor (rTumor), Fractional Anisotropy (FA). Area Under the Receiver Operating Characteristic curve (AUC).

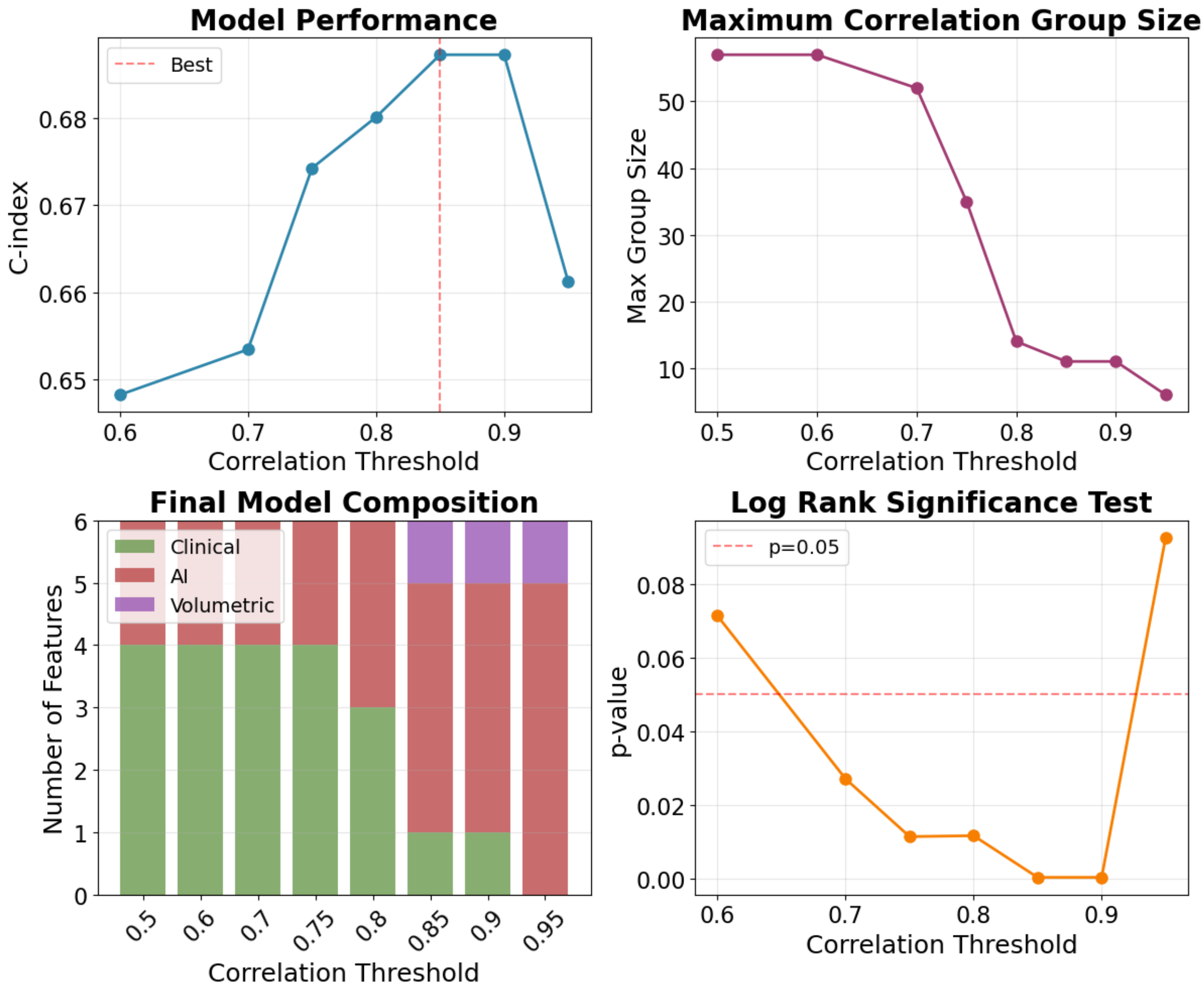

Supplemental Figure 6: Analysis of correlation threshold for grouping features for survival analysis. Pearson's correlation coefficient of 0.85 was chosen since it produced CoxPH model with the highest C-Index.

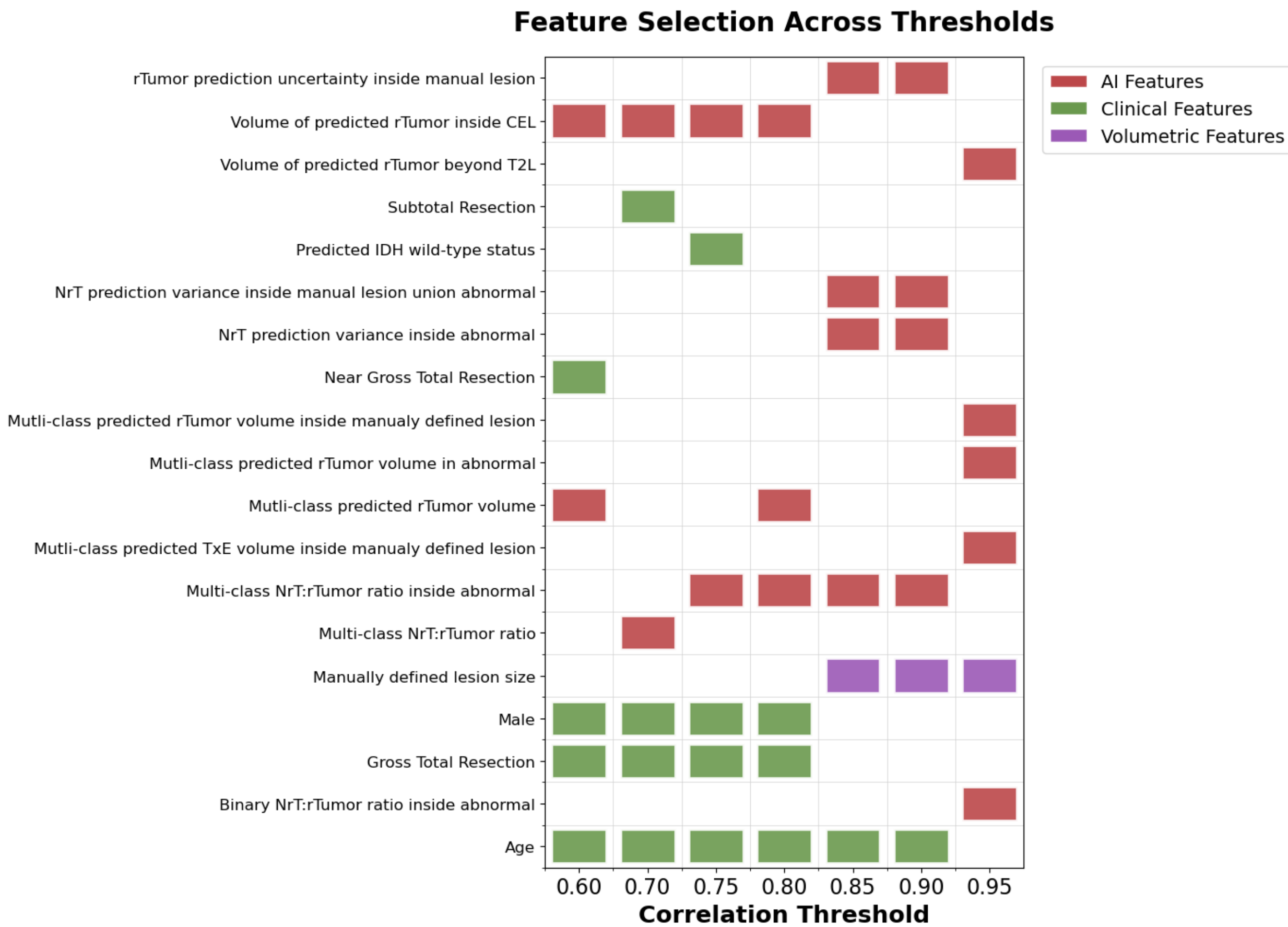

Supplemental Figure 7: Feature consistency through various correlation thresholds for grouping features.

Supplemental Table 1: Statistics for metrics used in survival analysis.

	Mean	Median	Min	Max	IQR	Count
**Age**	53.960	54.112	23.586	84.345	17.205	
**Ratio of NrT:rTumor in the T2L + CEL**	10.937	5.834	0.224	72.290	9.386	
**Ratio of NrT:rTumor in predicted abnormal**	4.651	3.541	0.538	20.259	3.708	
**Ratio of NrT:rTumor in predicted abnormal + T2L + CEL**	4.909	3.694	0.566	22.325	3.782	
**Ratio NrT:Total in predicted abnormal + T2L + CEL**	0.742	0.787	0.361	0.957	0.183	
**Ratio NrT:Total in predicted abnormal**	0.735	0.780	0.350	0.953	0.184	
**Ratio NrT:Total in T2L + CEL**	0.786	0.854	0.183	0.986	0.195	
**Predicted rTumor size in predicted abnormal + T2L + CEL**	40.152	30.604	2.953	207.577	33.247	
**Predicted rTumor size in predicted abnormal**	39.714	30.506	2.871	207.577	32.963	
**Predicted rTumor size in T2L + CEL**	22.396	12.093	0.721	175.763	27.679	
**Predicted NrT size in predicted abnormal + T2L + CEL**	110.706	101.722	15.533	281.066	75.120	
**Predicted NrT size in predicted abnormal**	104.895	98.056	15.492	279.825	70.627	
**Predicted NrT size in T2L + CEL**	72.773	60.903	1.597	208.207	59.959	
**Predicted abnormal size + T2L + CEL**	150.858	147.327	26.551	358.617	102.08	
**Predicted abnormal size**	144.609	134.557	26.509	358.478	96.451	
**Predicted NrT size in predicted abnormal beyond T2L + CEL**	38.079	32.206	9.179	113.691	34.641	
**Predicted rTumor size in predicted abnormal beyond T2L + CEL**	17.758	13.387	2.232	67.656	16.094	
**Volume of predicted abnormal beyond T2L + CEL**	55.837	49.572	13.079	155.500	44.641	
**Ratio NrT:rTumor in abnormal beyond T2L + CEL**	0.686	0.698	0.288	0.913	0.131	
**Average prediction value in predicted abnormal + T2L + CEL**	0.536	0.549	0.340	0.701	0.093	
**Average prediction value in predicted abnormal**	0.532	0.547	0.333	0.697	0.098	
**Average prediction value in T2L + CEL**	0.551	0.566	0.317	0.715	0.105	
**Multi-class predicted size of rTumor**	55.407	48.346	9.413	181.711	49.405	
**Multi-class predicted size of rTumor inside T2L + CEL**	13.090	7.181	0.454	95.375	14.595	
**Multi-class predicted size of rTumor inside predicted abnormal + T2L + CEL**	24.156	19.098	2.346	113.546	22.390	
**Multi-class predicted size of rTumor inside predicted abnormal**	24.156	19.098	2.346	113.546	22.390	
**Multi-class predicted size of NrT**	717.697	666.987	96.168	1336.881	372.10	
**Multi-class predicted size of NrT inside T2L + CEL**	75.716	65.639	2.862	206.103	66.871	
**Multi-class predicted size of NrT inside predicted abnormal + T2L + CEL**	120.452	113.158	19.261	302.160	77.716	
**Multi-class predicted size of NrT inside predicted abnormal**	120.452	113.158	19.261	302.160	77.716	
**Multi-class ratio NrT:rTumor**	21.122	13.884	2.076	110.338	17.270	
**Multi-class ratio NrT:rTumor inside T2L + CEL**	16.085	8.787	0.489	57.500	21.601	
**Multi-class ratio NrT:rTumor inside predicted abnormal + T2L + CEL**	8.265	6.315	1.014	31.549	7.226	
**Multi-class ratio NrT:rTumor inside predicted abnormal**	8.265	6.315	1.014	31.549	7.226	
**NrT uncertainty in predicted abnormal + T2L + CEL**	0.009	0.009	0.007	0.016	0.002	
**NrT uncertainty in predicted abnormal**	0.008	0.008	0.006	0.014	0.002	
**NrT uncertainty in T2L + CEL**	0.009	0.009	0.007	0.016	0.002	
**NrT variance in predicted abnormal + T2L + CEL**	0.004	0.004	0.003	0.008	0.001	

**NrT variance in predicted abnormal**	0.004	0.004	0.002	0.007	9.8E-04	
**NrT variance in T2L + CEL**	0.004	0.004	0.003	0.008	0.001	
**rTumor uncertainty in predicted abnormal + T2L + CEL**	0.008	0.008	0.007	0.012	0.001	
**rTumor uncertainty in predicted abnormal**	0.007	0.008	0.005	0.009	8.8E-04	
**rTumor uncertainty in T2L + CEL**	0.008	0.008	0.006	0.012	0.001	
**rTumor variance in predicted abnormal + T2L + CEL**	0.004	0.004	0.003	0.005	5.2E-04	
**rTumor variance in predicted abnormal**	0.003	0.003	0.002	0.004	4.9E-04	
**rTumor variance in T2L + CEL**	0.004	0.004	0.002	0.005	5.7E-04	
**Average variance in predicted abnormal + T2L + CEL**	0.004	0.004	0.003	0.006	5.7E-04	
**Average variance predicted abnormal**	0.004	0.004	0.003	0.005	5.5E-04	
**Average variance in T2L + CEL**	0.004	0.004	0.003	0.007	7.7E-04	
**Average uncertainty in predicted abnormal + T2 + CEL**	0.009	0.008	0.007	0.013	0.001	
**Average uncertainty in predicted abnormal**	0.009	0.008	0.007	0.012	9.8E-04	
**Average uncertainty in T2L + CEL**	0.009	0.008	0.006	0.014	0.001	
**T2L size**	91.888	90.191	7.868	252.226	81.263	
**CEL size**	14.415	11.906	0.099	48.445	16.784	
**T2L + CEL size**	95.169	91.223	8.066	252.375	81.947	
**rTumor size in T2L**	21.982	11.232	0.705	175.714	26.622	
**NrT size in T2L**	69.906	58.183	1.384	206.624	60.608	
**rTumor size in CEL**	1.858	0.784	0.000	15.242	2.936	
**NrT size in CEL**	12.556	11.052	0.055	42.656	12.795	
**Predicted IDH wild-type**	-	-	-	-	-	47
**Gross Total Resection**	-	-	-	-	-	13
**Subtotal Resection**	-	-	-	-	-	32
**near Gross Total Resection**	-	-	-	-	-	11
**Male**	-	-	-	-	-	37
**Survival Days**	330	288	39	985	260	

CEL = Contrast enhancing lesion
T2L = T2-hyperintense lesion
NrT = Non-recurrent tumor
rTumor = recurrent tumor
IDH = isocitrate dehydrogenase

Supplemental Table 2: Label distribution for training and testing, for both repeated constrained cross validation (RCCV) nested testing and using 5 disjoint test sets with repeated, grouped, and stratified, cross validation only on the training sets (with 5 independent tuning rounds determined the hyperparameter set for each round of testing).

**Nested testing with repetitions (RCCV)**														
**Split**	**Train Total**	**Train NrT**	**Train %NrT**	**Train rTumor**	**Train %rTumor**	**Train NAB**	**Train %NAB**	**Test Total**	**Test NrT**	**Test %NrT**	**Test rTumor**	**Test %rTumor**	**Test NAB**	**Test %NAB**
**1**	503	58	11.53	140	27.83	305	60.64	146	17	11.64	39	26.71	90	61.64
**2**	502	61	12.15	133	26.49	308	61.35	147	14	9.52	46	31.29	87	59.18
**3**	501	53	10.58	142	28.34	306	61.08	148	22	14.86	37	25.0	89	60.14
**4**	510	63	12.35	136	26.67	311	60.98	139	12	8.63	43	30.94	84	60.43
**5**	499	56	11.22	138	27.66	305	61.12	150	19	12.67	41	27.33	90	60.0
**6**	501	57	11.38	139	27.74	305	60.88	148	18	12.16	40	27.03	90	60.81
**7**	511	63	12.33	133	26.03	315	61.64	138	12	8.7	46	33.33	80	57.97
**8**	500	56	11.2	139	27.8	305	61.0	149	19	12.75	40	26.85	90	60.4
**9**	499	54	10.82	140	28.06	305	61.12	150	21	14.0	39	26.0	90	60.0
**10**	501	63	12.57	133	26.55	305	60.88	148	12	8.11	46	31.08	90	60.81
**11**	496	59	11.9	132	26.61	305	61.49	153	16	10.46	47	30.72	90	58.82
**12**	504	57	11.31	138	27.38	309	61.31	145	18	12.41	41	28.28	86	59.31
**13**	502	61	12.15	133	26.49	308	61.35	147	14	9.52	46	31.29	87	59.18
**14**	509	58	11.39	143	28.09	308	60.51	140	17	12.14	36	25.71	87	62.14
**15**	501	59	11.78	137	27.35	305	60.88	148	16	10.81	42	28.38	90	60.81
**16**	499	60	12.02	134	26.85	305	61.12	150	15	10.0	45	30.0	90	60.0
**17**	509	55	10.81	142	27.9	312	61.3	140	20	14.29	37	26.43	83	59.29
**18**	498	63	12.65	130	26.1	305	61.24	151	12	7.95	49	32.45	90	59.6
**19**	506	63	12.45	135	26.68	308	60.87	143	12	8.39	44	30.77	87	60.84
**20**	498	58	11.65	135	27.11	305	61.24	151	17	11.26	44	29.14	90	59.6
**Disjoint Test sets**														
**Split**	**Train Total**	**Train NrT**	**Train %NrT**	**Train rTumor**	**Train %rTumor**			**Test Total**	**Test NrT**	**Test %NrT**	**Test rTumor**	**Test %rTumor**		
**1**	199	67	33.7	132	66.3			55	8	14.5	47	85.5		
**2**	212	61	28.8	151	71.2			42	14	33.3	28	66.7		
**3**	201	60	29.9	141	70.1			53	15	28.3	38	71.7		
**4**	198	53	26.8	145	73.2			56	22	39.3	34	60.7		
**5**	206	59	28.6	147	71.4			48	16	33.3	32	66.7		
